# Replacing American Breakfast Foods with Ready-To-Eat (RTE) Cereals Increases Consumption of Key Food Groups and Nutrients among US Children and Adults: Results of an NHANES Modeling Study

**DOI:** 10.3390/nu9091010

**Published:** 2017-09-13

**Authors:** Colin D. Rehm, Adam Drewnowski

**Affiliations:** Center for Public Health Nutrition, University of Washington, Seattle, WA 98195-3410, USA; crehm@uw.edu

**Keywords:** breakfast, nutrient density, diet quality, diet cost, modeling

## Abstract

Replacing the typical American breakfast with ready-to-eat cereals (RTECs) may improve diet quality. Our goal was to assess the impact of RTECs on diet quality measures for different age groups, using substitution modeling. Dietary intakes came from the 2007–2010 National Health and Examination Surveys (NHANES; *n* = 18,112). All breakfast foods, excluding beverages, were replaced on a per calorie basis, with frequency-weighted and age/race specific RTECs. Model 1 replaced foods with RTECs alone; Model 2 replaced foods with RTECs and milk. Diet quality measures were based on desirable food groups and nutrients, Healthy Eating Index (HEI)-2010 scores, and estimated diet costs. Model 1 diets were significantly higher in whole grains (+84.6%), fiber (+14.3%), vitamin D (+14.0%), iron (+54.5%) and folic acid (+104.6%), as compared to observed diets. Model 2 diets were additionally higher in dairy (+15.8%), calcium (+11.3%) and potassium (+3.95%). In Model 1, added sugar increased (+5.0%), but solid fats declined (−10.9%). Energy from solid fats and added sugars declined (−3.2%) in both models. Model 2 offered higher diet quality (57.1 vs. 54.6, *p*-value < 0.01) at a lower cost ($6.70 vs. $6.92; *p* < 0.01), compared to observed diets. Substitution modeling of NHANES data can assess the nutritional and economic impact of dietary guidance.

## 1. Introduction

Often referred to as the most important meal of the day, breakfast represents an important source of key nutrients in the American diet [1,2,3,4]. Eating breakfast is associated with higher diet quality [1,5], and with an improved nutrient adequacy [2,4,6]. Eating breakfast, as opposed to skipping it, has been associated with a lower likelihood of being obese, and with lower risks of diabetes and metabolic disease [4,7,8,9,10,11]. Though a causal link between breakfast and lower body weight has not been established [12,13], eating breakfast on a regular basis may be a valuable aid in weight control [14]. Consumption of whole grain cereals was also found to be associated with a reduced risk of heart failure in a dose–response manner [15].

Ready-to-eat cereals (RTECs) were featured prominently among 12 breakfast patterns, based on NHANES 2001–2008 data [1]. While 19% of the population skipped breakfast altogether, about 37% consumed grain products, alone or with fruit juice. Other breakfast patterns included eggs, meat, poultry and fish, sweets, cooked cereal, whole fruit and coffee or tea. About 16% of the population consumed RTECs, more often with low-fat than with whole milk [1]. RTEC consumers had higher intakes of shortfall nutrients and lower intakes of nutrients to limit, than did the breakfast skippers [1]. In other studies also, RTEC consumption was associated with higher quality diets [16,17].

The present study used substitution modeling to examine the contribution of RTECs to nutrient intakes and overall diet quality, in the NHANES 2007–2010 population, by age group. Following prior studies [18,19,20], we developed a new method to evaluate the nutritional impact of replacing solid foods eaten by Americans at breakfast with a composite RTEC package. The RTEC package was based on the usual patterns of RTEC consumption by different age-race groups from the NHANES database. Solid foods eaten at breakfast (but not beverages) were replaced, in isocaloric amounts, with those RTECs that were most frequently eaten by that age-race group. Assessments of dietary nutrient density were based on food groups and nutrients of public health concern (e.g., whole grains, fiber, calcium, vitamin D), and on food groups and nutrients to limit (e.g., solid fats, added sugars and sodium). Overall diet quality was measured using Healthy Eating Index (HEI) 2010 scores [21]. Given that RTECs are among the lowest-cost sources of some key nutrients [22], further analyses assessed resulting diet costs using the United States Department of Agriculture (USDA) national food prices database, adjusted for inflation [23].

## 2. Materials and Methods

### 2.1. Study Population and Dietary Data

Data for this study were drawn from the nationally representative National Health and Nutrition Examination Survey (NHANES). Data from the 2007–2008 and 2009–2010 cycles of NHANES were used. At the time of study initiation (Winter 2014), the 2007–2008 and 2009–2010 cycles were the most current NHANES data with dietary data. Data were available for 18,112 children, adolescents and adults aged ≥ 1 years. Any children who consumed breast milk were excluded. The sample included 6930 children and adolescents (aged 1–19 years) and 11,182 adults (aged ≥ 20 years). We opted to include children aged 1–2 years, as cereal is frequently consumed in this age group.

The source of dietary data for all analyses, other than those of the Healthy Eating Index-2010 score, was two non-consecutive 24-h recalls. The first 24-h recall was completed in-person at the Mobile Examination Center with a trained interviewer. The second recall was completed over the telephone many days later. The 24-h recall queries all foods/beverages consumed by participants from midnight-to-midnight on the previous day [24]. The NHANES 24-h recall also collects information on where the food/beverages were obtained, the time they were consumed and the participant-reported name of the eating occasion (i.e., breakfast, lunch, dinner, snack, beverage). Eighty-five percent of respondents completed two dietary recalls and the remaining 15% completed a single dietary recall.

This information was supplemented with data from the Food Patterns Equivalents Database (FPED), developed by the United States Department of Agriculture (USDA). In addition, to evaluate any economic implications of replacing ready-to-eat cereals with other breakfast items, estimates of the cost of every food item were obtained. The national food prices database was initially developed by the Center for Nutrition Policy (CNPP) at the USDA for 2001–2002 and 2003–2004, providing the price per 100 grams of edible portions, for all consumed foods in the NHANES, exclusive of alcoholic beverages, baby foods and infant formulas [25,26]. Accounting for food group specific price changes from 2003–2004 to 2010, we have updated the prices for inflation using a procedure described elsewhere [23].

### 2.2. Modeling Strategy

The present goal was to evaluate the impact of replacing breakfast foods with RTECs. Meal descriptions were collected as part of the NHANES 24-h recall. Breakfast foods were defined as those foods that were reported as being eaten at breakfast, “desayuno” or brunch. Only solid foods were replaced; beverages consumed at breakfast (e.g., milk or orange juice) were not.

RTECs were defined as grain-based products, primarily consumed at breakfast, that require no heating or preparation. Excluded from this definition were hot cereals (e.g., oatmeal or grits) and cereal-based snack bars (e.g., granola bars). This definition was operationalized based on the food codes within the Food and Nutrient Database for Dietary Studies (FNDDS)—specifically eight-digit food codes with the prefixes 571, 572, 573, and 574. The five most commonly consumed cereals in the NHANES sample were Cheerios, Honey Nut Cheerios, Frosted Flakes, Fruit Loops and Frosted Mini-Wheats.

The modeled cereals were defined by evaluating age/race-specific consumption of all ready-to-eat cereals. Eight age groups (1–3 years, 4–8 years, 9–13 years, 14–19 years, 20–30 years, 31–50 years, 51–70 years, and ≥71 years) and five race/ethnicity groups (non-Hispanic white, non-Hispanic black, Mexican-American, other Hispanic and other/mixed race) were used to create the model RTECs. These age groups roughly matched those used by the Institute of Medicine in determining Dietary Reference Intakes (DRIs). Because of this, the nutrient composition of the composite RTECs varied by population sub-group. The composite RTECs for children included more sweetened cereals, whereas those for adults tended to include more unsweetened and whole grain cereals. For example, among 9–13 years old non-Hispanic white children, Cinnamon Toast Crunch, Honey Nut Cheerios, Lucky Charms, Cocoa Puffs, and Fruity Pebbles each contributed more than 5%, and collectively accounted for 26%, of the composite RTEC profile. For 51–70 years old non-Hispanic white adults, Cheerios, Honey Nut Cheerios, Frosted Mini-Wheats (all flavors), Raisin Bran, Granola (not further specified), and Honey Bunches of Oats with Almonds each received more than 4% weight, and collectively accounted for 35% of the composite RTEC profile.

The composite RTECs were based on all RTECs eaten during the day, and so reflected total RTEC consumption. About 76% of RTECs were consumed at breakfast. For children, adolescents and younger adults (20–30 years), cereals eaten at breakfast were similar to those eaten outside of breakfast in terms of nutrient density, and whole grain and added sugar content. For older adults (age > 30 years), cereals consumed at breakfast contained fewer added sugars and more whole grains. Supplemental Tables S1–S41 describe component cereals contributing more than 1% of the total RTEC weight for each age-race/ethnicity subpopulation.

Two models were developed, one that used only RTECs and another using a combination of RTECs and milk. Model 1 replaced solid foods consumed at breakfast with RTECs on a calorie-per-calorie basis, whereas Model 2 replaced solid breakfast foods with RTECs and milk, also on a calorie-per-calorie basis. Beverages and beverage additions (i.e., sugar added to coffee) were not replaced and the diets of individuals already consuming RTECs were not modified. For example, an individual consuming a cappuccino and a doughnut would have the calories from the doughnut replaced with RTECs (with or without milk), but not the calories from the cappuccino. An individual consuming only a beverage at breakfast would not have any energy replaced.

Model 2 included the replacement of breakfast foods with a combination of RTEC and milk. The type and average amount of milk added to cereal was determined by the age/race-specific use of milk, in a similar manner to the cereal replacement. Only milk that was consumed with cereal was included when deriving the proportion of people adding milk to cereal and the amount of milk added. Like cereal, the type of milk used with cereal varied by population sub-group—younger children were more likely to consume cereal with higher fat milks, as were Mexican-American, other Hispanic and non-Hispanic black children. On the other hand, older adults were more likely to consume cereal with non-fat and low-fat milk. Children were more likely to consume cereal without milk, than adults.

The primary outcome measures were based on either food groups or nutrients of interest. Their selection was guided by the current dietary recommendations [16,27,28]. For example, fiber, vitamin D, calcium, magnesium, and potassium were all identified in the 2010 Dietary Guidelines for Americans as nutrients of concern [27]. Food groups of interest included whole grains, refined grains, and dairy. We also considered the impact of substitution modeling on solid fat and added sugars, collectively and individually. Three nutrients of concern for specific population sub-groups were also examined: iron (for adolescent girls and women capable of becoming pregnant), folic acid (for women capable of becoming pregnant) and vitamin B12 (for older adults) [27].

Finally, we examined the impact of substitution modeling on the 2010 Healthy Eating Index [20] scores. The 2010 Healthy Eating Index (HEI 2010) measures adherence to the 2010 Dietary Guidelines for Americans based on consumption of nine food groups/nutrients to encourage (i.e., total vegetables, dark-green and orange vegetables, total fruit, whole fruit, whole grains, total protein foods, protein from seafood and plant sources, the ratio of polyunsaturated and monounsaturated fatty acids to saturated fatty acids, and total dairy) and three food groups/nutrients to discourage (refined grains, sodium, and a combined measure of added sugars, solid fat, and alcohol—a summary measure of empty calories). The HEI-2010 score is an energy-adjusted diet quality score [21]. Analyses of the HEI-2010 score were limited to the first 24-h recall. Finally, the impact of substitution modeling on the estimated diet costs was evaluated using two dietary recalls and national food prices, adjusted for inflation.

### 2.3. Analyses

The National Cancer Institute (NCI) method was used to characterize the usual intake of nutrients and food groups of interest [29,30,31]. This method can be used to estimate the usual intake of nutrients and food groups, including the population distribution of intakes. Two models were fit using this method, one for ubiquitously consumed nutrients or food groups (i.e., foods/nutrients consumed by most individuals on all days, such as fiber or refined grains) and another, which incorporated both the mean and probability of consumption for episodically consumed foods/nutrients (i.e., not consumed by most individuals on all days, such as whole grains). Additional covariates were included in the model to account for whether the recall data were from a weekday or weekend, and whether it was the first or second recall, and accounted for mode (e.g., telephone vs. in-person) and order effects. Estimates of the population mean and standard error and distribution of intakes were conducted for observed diets and for Models 1 and 2, for the entire population and by age group. Additional analyses were conducted for children who participated in the Women, Infants and Children (WIC) nutrition program in the past year, though the sample size was insufficient for some episodically consumed foods (e.g., whole grains). There were insufficient data to evaluate the dietary intakes of women participating in the WIC program.

In order to account for the complex NHANES survey design, balanced repeated replication (BRR) weights were constructed using WesVar software (Westat, Rockville, MD, USA, 2012) and a Fay’s adjustment of 0.7. A total of 32 BRR runs were repeated for each analysis, making the results representative of the United States of America (US) population. While nutrients and specific food groups were evaluated using the NCI method, the HEI-2010 was estimated using the population ratio method, using data from the first 24-h recall [30]. To determine if mean intakes differed for the different models, as compared to the observed values, we conducted survey-weighted *t*-tests with an unequal variance. To place the results in context, we defined a relative change of less than 5% to be “marginal”, if the difference was statistically significant (*p* < 0.05). Statistically significant differences (*p* < 0.05), for which the relative change was between 5% and 10% (or between −5% and −10%), are described as “modest” or “moderate” changes, while statistically significant changes greater than 10% (or less than −10%), are described as “strong” or “dramatic”. Ten percent was selected as the cut-off for “strong” effects, as it corresponds to the definition of a “good source” of nutrients or minerals, according to the US Food and Drug Administration [32]. All output for this paper was generated using SAS software, Version 9.3 and are representative of the US population (SAS Institute Inc., Cary, NC, USA).

### 2.4. Data Availability and Ethical Approval

The necessary Institutional Review Board (IRB) approval for NHANES had been obtained by the National Center for Health Statistics (NCHS) [33]. For adult participants, written informed consent was obtained directly from the participating adult. For child participants, parental/guardian written informed consent was obtained and children/adolescents aged ≥ 12 years provided additional written consent. All data used here are publicly available on the NCHS and USDA websites [34,35]. Publicly available data, such as those used here, per University of Washington policies, do not involve “human subjects” and their use requires neither IRB review nor an exempt determination. According to University of Washington policies, these data may be used without any involvement of the Human Subjects Division or the University of Washington IRB.

## 3. Results

Among the 18,112 study participants, 83.0% consumed breakfast (defined as energy from any foods/beverages at breakfast). After excluding individuals who only consumed beverages at breakfast and individuals consuming any RTEC at breakfast, 41.6% (*n* = 7365) consumed food that was eligible for substitution. After excluding energy from beverages, as beverages were not eligible for replacement, the mean and median energy at breakfast were 367 and 312 kcal, respectively. Breakfast energy to be replaced varied by age, as follows: 1–3 years (234 kcal); 4–8 years (303 kcal); 9–13 years (379 kcal); 14–19 years (392 kcal); 20–30 years (403 kcal); 31–50 years (395 kcal), 51–70 years (351 kcal), and ≥71 years (325 kcal). This was the amount of energy included in the replacement models. For Cheerios and Frosted Mini-Wheats (two of the most frequently consumed RTECs), the median value of 312 kcal corresponds to 85 g and 91 g without milk, and 71 g and 76 g if consumed with a half-cup of low-fat milk, respectively.

**Figure 1:**
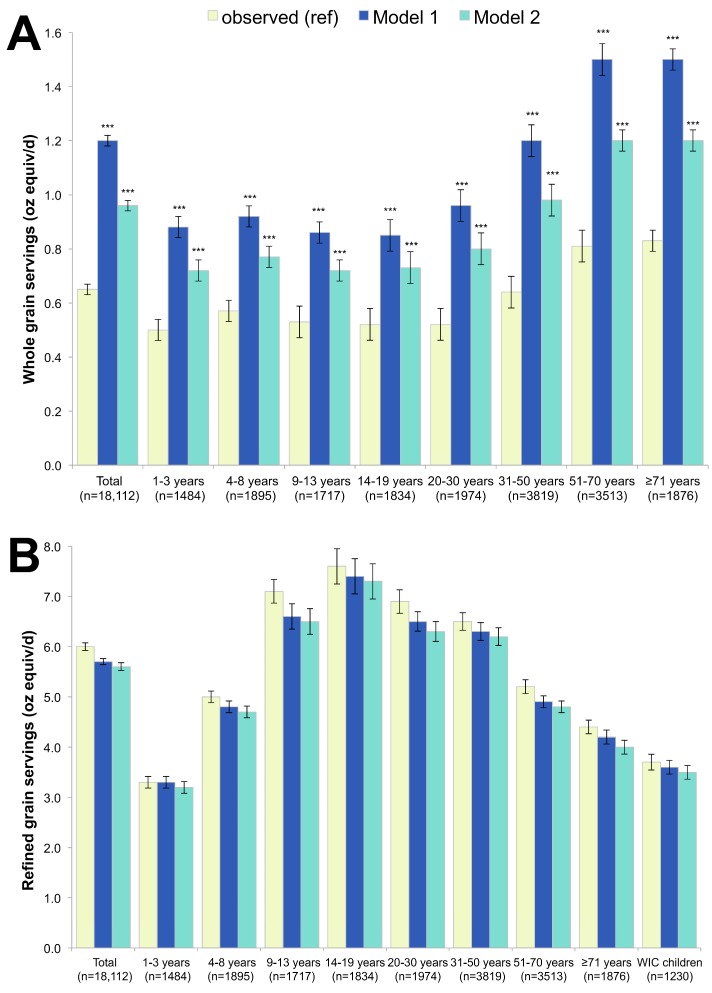
Whole grains (**A**) and refined grains (**B**) observed and in replacement models using ready-to-eat cereal (RTEC) alone (Model 1) and RTEC plus milk (Model 2). Model 1 represents substitution with ready-to-eat-cereals (RTEC) and Model 2 represents substitution with RTEC and milk. The *p*-values of the differences between each model and observed values are indicated by asterisk (*** *p* < 0.001). Error bars are 95% confidence intervals. WIC stands for Women, Infants and Children and indicates participation in this supplemental nutrition program.

Whole grain consumption increased dramatically (≥10% increase) from the observed mean of 0.65 ounce equivalents per day to 1.16 ounce equivalents per day in Model 1 (RTEC only) and to 0.96 ounce equivalents in Model 2 (RTEC + milk), a significant increase from the observed diets (*p* < 0.001 for both) (see Figure 1A). The strong and significant increase in whole grain consumption in both models held for all age groups. While the population’s average whole grain consumption increased by 85% and 48% in Models 1 and 2, respectively, the percent of the population consuming recommended amounts of whole grains remained quite low. For example, 3.0 ounce equivalents per day are recommended for both 9–13 year-old boys, and girls, but even after applying the models, the 90th percentile of whole grain consumption was only 1.30 and 1.12 for Models 1 and 2, respectively, compared to the 0.98 ounce equivalents observed. Based on observed food patterns, whole grains account for only 9.8% of total grains. The contribution of whole grains to total grains increased to 17.4% in Model 1 and 14.4% in Model 2. As shown in Figure 1, refined grain consumption was unchanged.

**Figure 2:**
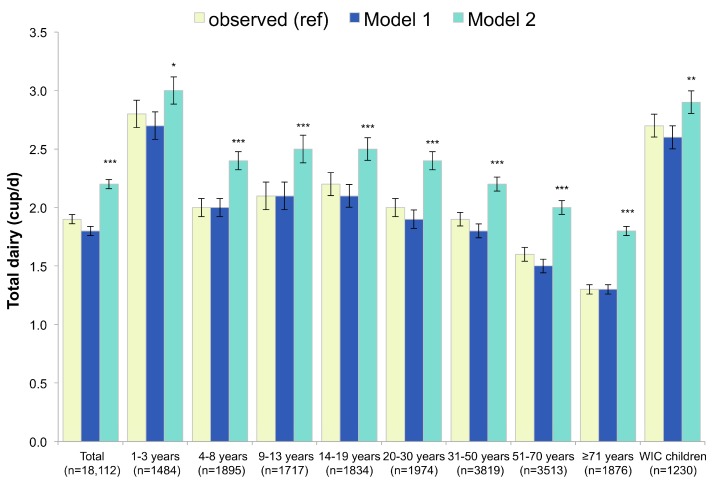
Total dairy in observed diets and in replacement models using RTEC alone (Model 1) and RTEC plus milk (Model 2). Model 1 represents substitution with ready-to-eat-cereals (RTEC) and Model 2 represents substitution with RTEC and milk. The *p*-values of the differences between each model and observed values are indicated by asterisk (*** *p* < 0.001; ** 0.001 < *p*-value < 0.01; * 0.05 < *p* < 0.01). WIC stands for Women, Infants and Children and indicates participation in this supplemental nutrition program.

An additional food group of interest was total dairy (i.e., dairy from all sources, including milk, yogurt and cheese). As shown in Figure 2, total dairy consumption did not change in Model 1, as compared to the observed diets. Dairy consumption increased strongly in Model 2. The strength of the modeled increase in dairy held for all age groups, but was of moderate strength for 1–3 year-old and children participating in WIC (increase between 5% and 9.99%). Three cups of dairy a day are recommended for boys and girls aged 9–13 years. As observed, and in Model 1, 12.3% and 11.8% of this age group met the recommendation, as compared to 19.6% in Model 2.

**Figure 3:**
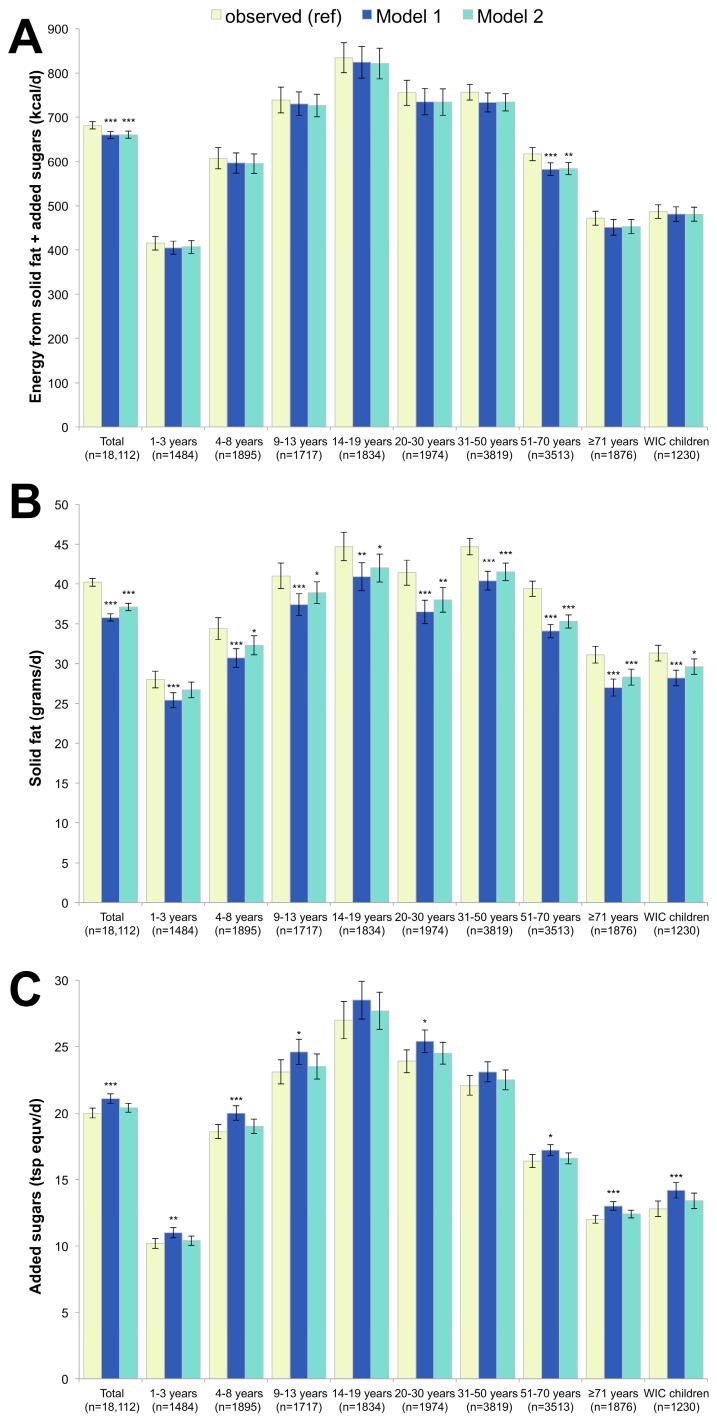
Energy from solid fats and added sugars (**A**), grams of solid fat (**B**) and teaspoons of added sugars (**C**) in observed diets and in replacement models using RTEC alone (Model 1) and RTEC plus milk (Model 2). Model 1 represents substitution with ready-to-eat-cereals (RTEC) and Model 2 represents substitution with RTEC and milk. The *p*-values of the differences between each model and observed values are indicated by asterisk (*** *p* < 0.001; ** 0.001 < *p*-value < 0.01; * 0.05 < *p* < 0.01). WIC stands for Women, Infants and Children and indicates participation in this supplemental nutrition program.

The 2010 Dietary Guidelines for Americans (DGAs) identified solid fats and added sugars as the main sources of empty calories in the US diet. Figure 3 shows that replacing breakfast foods with RTECs did not result in increases in combined solid fat and added sugar consumption. For energy from solid fats and added sugars overall, there was a significant marginal decrease between the observed intakes (682 kcal/day) and Models 1 and 2 (660 kcal/day for both). A significant, but modest decrease was also observed among adults aged 51–70 years (−5.7% and −5.5% in Models 1 and 2, respectively).

Overall, Model 1 resulted in a statistically significant and strong 10.9% decrease in the consumption of solid fats, while Model 2 resulted in moderate declines in solid fat consumption (a 7.7% decrease). While solid fats were reduced either dramatically or modestly in most age groups for Model 2, they were unchanged among children aged 1–3 years, due to the greater proportion of children eating RTECs with whole/reduced-fat milk, as opposed to skim/low-fat milk. Also shown in Figure 3, is the impact of the models on added sugar consumption. Model 1 led to a modest, but statistically significant increase in added sugars (a 5.5% increase from 20.0 to 21.1 teaspoon equivalents/day). The increases were of moderate strength for children and older adults. Model 2 resulted in no change in added sugars.

**Figure 4:**
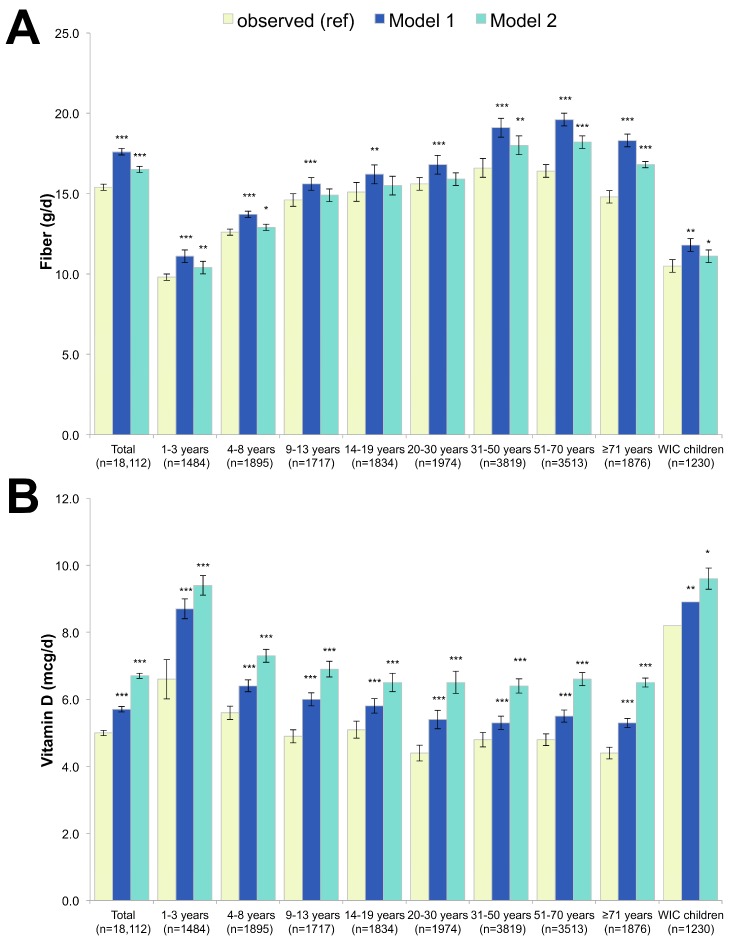

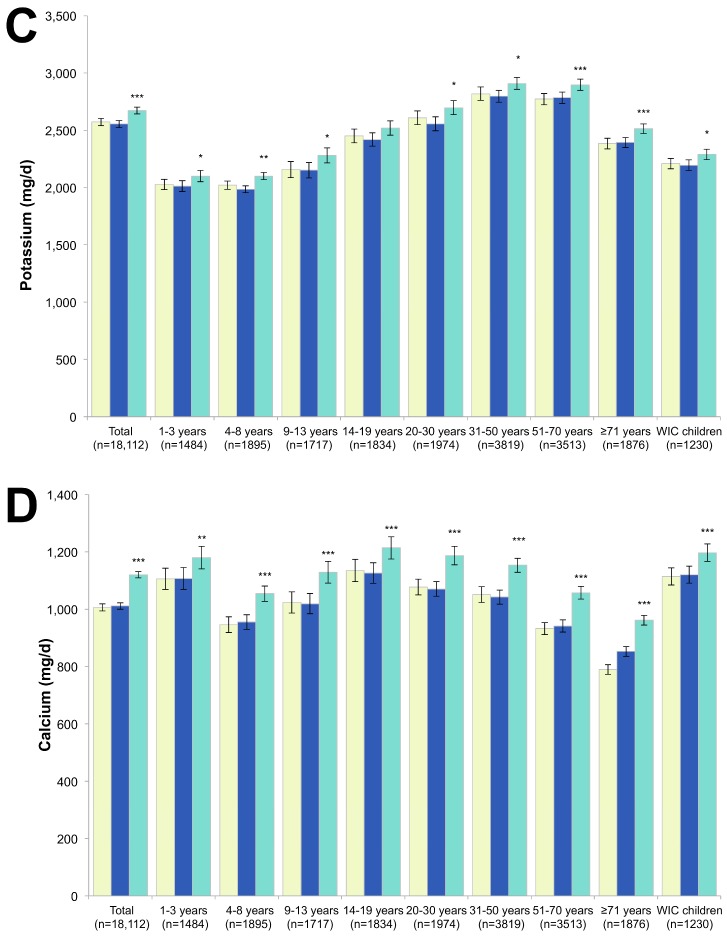
Dietary fiber (**A**), vitamin D (**B**), potassium (**C**) and calcium (**D**) in observed diets and in replacement models using RTEC alone (Model 1) and RTEC plus milk (Model 2). Model 1 represents substitution with ready-to-eat-cereals (RTEC) and Model 2 represents substitution with RTEC and milk. The *p*-values of the differences between each model to observed values are indicated by asterisk (*** *p* < 0.001; ** 0.001 < *p*-value < 0.01; * 0.05 < *p* < 0.01). WIC stands for Women, Infants and Children and indicates participation in this supplemental nutrition program.

Figure 4 shows that the substitution of breakfast foods with RTEC led to dramatic increases in dietary fiber intake in Model 1, increasing from 15.4 g/day to 17.6 g/day, a 14% increase. In Model 2, the fiber effects were more modest (a 7.1% increase to 16.5 g/day). The proportion of individuals consuming more than 25 g of dietary fiber a day increased from 7.1% to 13.2% for Model 1 and 9.6% for Model 2. Vitamin D dramatically increased in both models and among all age groups (+14% in Model 1 and +34% in Model 2). Overall, effects of the models on potassium consumption were marginal, but potassium was increased modestly among 9–13 year-old (+5.7%) and among the oldest adults (+5.4%) in Model 2. In Model 1, no change was observed for calcium overall, though the oldest adults had a modest increase (+8.0%). In Model 2, a dramatic increase was observed for calcium (+11.3%), with the effects varying by age group.

**Figure 5:**
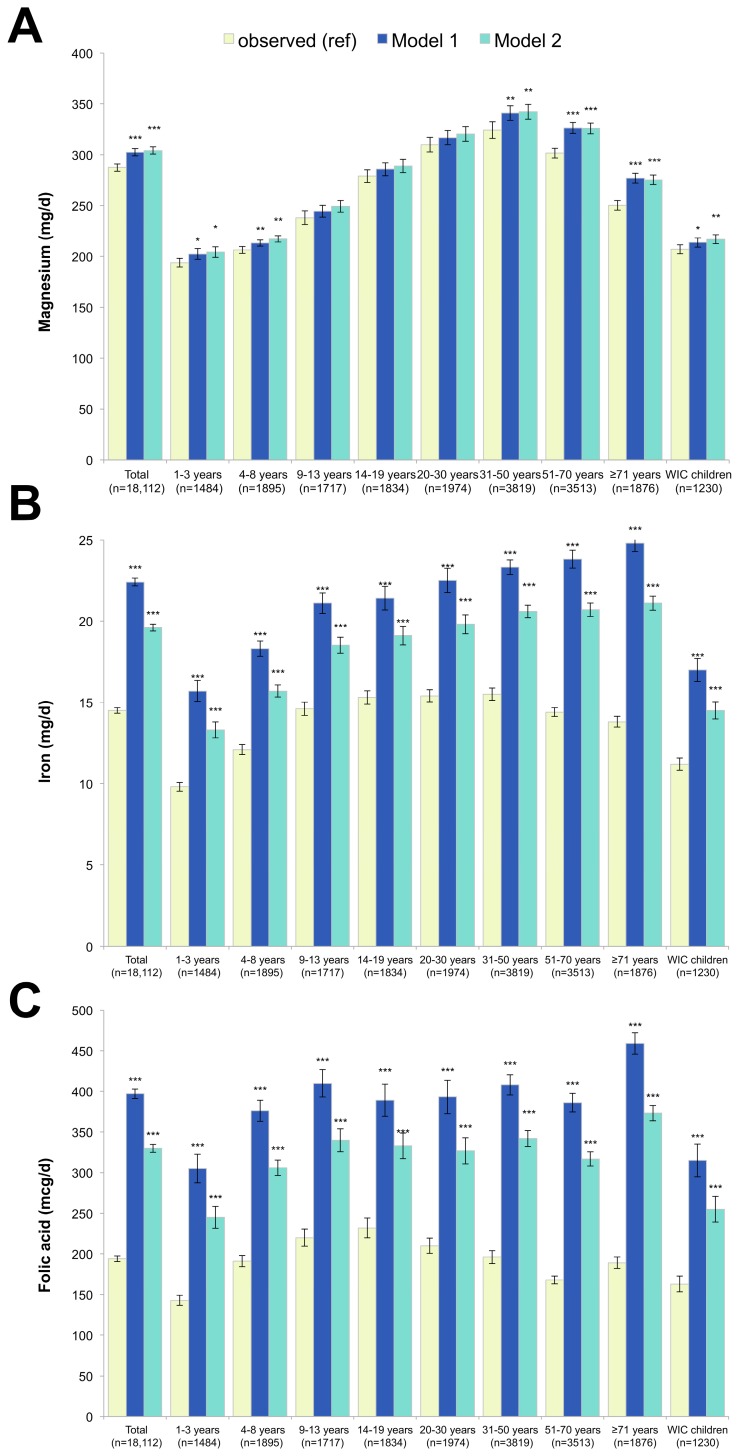
Magnesium (**A**), iron (**B**) and folic acid (**C**) in observed diets and in replacement models using RTEC alone (Model 1) and RTEC plus milk (Model 2). Model 1 represents substitution with ready-to-eat-cereals (RTEC) and Model 2 represents substitution with RTEC and milk. The *p*-values of the differences between each model and observed values are indicated by asterisk (*** *p* < 0.001; ** 0.001 < *p*-value < 0.01; * 0.05 < *p* < 0.01). WIC stands for Women, Infants and Children and indicates participation in this supplemental nutrition program.

Figure 5 shows that magnesium was modestly increased in both Model 1 (+5.3%) and Model 2 (+5.9%), with the strongest effects observed amongst the oldest adults. In Model 1, iron dramatically increased by more than 50% and folic acid by more than 100%. Weaker, but still strong, effects for iron and folic acid were observed in Model 2. Among girls aged 14–19 years, the Recommended Daily Allowance (RDA) for iron is 15 mg/day. In the observed diets, 29.5% met the iron DRI, compared to 64.3% in Model 1 and 54.3% in Model 2. For women aged 19–50 years, the RDA is 18 mg/day; in the observed diets, 10.8% met the iron RDA, while 51.6% and 37.1% met the iron RDA in Models 1 and 2, respectively. For vitamin B12 (data not shown), among adults aged ≥71, intakes increased dramatically from 4.6 μgm/day to 7.9 and 7.4 μgm/day in Models 1 and 2, respectively.

**Figure 6:**
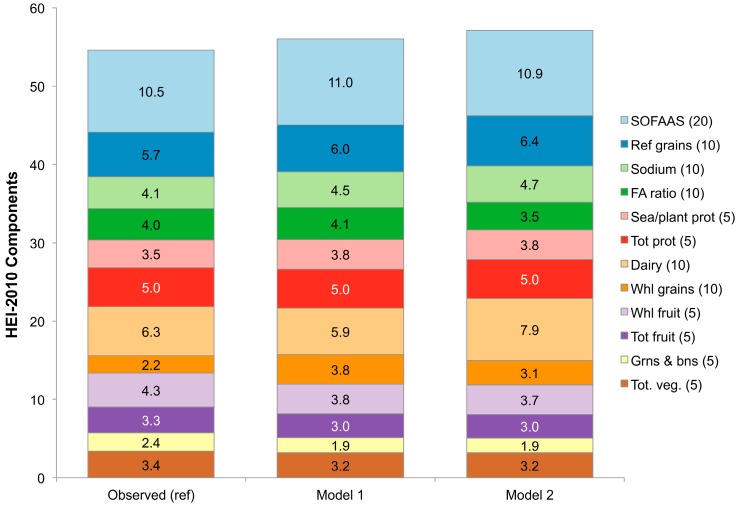
HEI-2010 ^a^ component scores under different modeling scenarios in the US population, 2007–2010. ^a^ The HEI-2010 is a 100-point score ranging from 0 to 100, which measures adherence to the 2010 Dietary Guidelines for Americans. A score of 100 indicates perfect adherence to the 2010 Dietary Guidelines for Americans. Higher scores indicate higher quality diets. The values in parentheses are the maximum values for each HEI-2010 component. SOFAAS stands for solid fats, alcohol and added sugars, and is a summary measure of empty calories.

The Healthy Eating Index-2010 is a summary measure of adherence to the 2010 Dietary Guidelines for Americans, serving as an index of overall diet quality [21]. Total HEI-2010 scores increased from the observed value of 54.6 to 57.1 in Model 2 (see Table 1), a statistically significant, but marginal effect. Overall, Model 1 (RTEC alone) did not lead to any change in the HEI-2010, but a marginal increase was observed among children aged 1–3 years. Both models led to higher HEI 2010 scores among adults aged 51–70 years. Figure 6 shows the impact of RTEC substitution on HEI 2010 component scores. Much of the change in total HEI-2010 scores was due to the higher consumption of whole grains (Models 1 and 2), lower consumption of empty calories from solid fats and added sugars (Models 1 and 2), and higher consumption of dairy products (Model 2 only).

**Figure 7:**
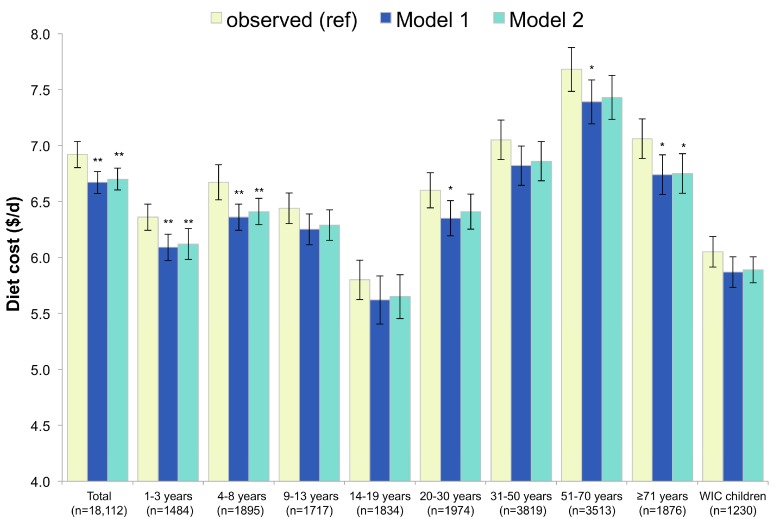
Diet cost as observed and in replacement models using RTEC alone (Model 1) and RTEC plus milk (Model 2). Model 1 represents substitution with ready-to-eat-cereals (RTECs) and Model 2 represents substitution with RTECs and milk. The *p*-Values of the differences between each model and observed values are indicated by asterisk (** 0.001 < *p*-value < 0.01; * 0.05 < *p* < 0.01). WIC stands for Women, Infants and Children and indicates participation in this supplemental nutrition program.

These increases in dietary nutrient density and modeled improvement in overall diet quality were achieved without a corresponding increase in estimated daily diet costs. Diet costs were marginally reduced in both Models 1 and 2, with the strongest effects observed among children (see Figure 7).

## 4. Discussion

The 2015 Dietary Guidelines Committee Advisory Report noted that the US diet does not meet recommendations for whole grains or dairy, and exceeds the guidelines for refined grains, solid fats and added sugars [36]. The MyPlate graphic of the USDA has long adopted the slogan: “Make half your grains whole”. In reality, whole grains account for less than 10% of total grain consumption, as opposed to 50%. One common recommendation is to substitute a whole grain product for a refined grain product [37]. This study took the concept of substitution several steps further, with the goal of modeling the nutritional and economic impact of replacing regular breakfast foods with RTECs (with or without milk) on the diets of US children, adolescent and adults.

While substitution modeling has been used previously to create optimal food patterns, the present methodology included some major innovations [18,19,20]. First, the substitutions were based on actual RTEC consumption patterns by age and race. The nutrient composition of RTECs was weighted by the relative frequency of consumption of specific RTECs by each age and race group. Second, Model 1 was based on RTECs alone, whereas Model 2 was based on RTECs and milk, where the type and amount of milk was also based on observed population patterns. Third, the substitution was sensitive to eating occasion and culture; RTECs are a common breakfast food in the US across a wide range of population sub-groups. The MyPlate recommendation to “make half your grains whole” incudes the advice to consume more bulgur, barley, buckwheat, whole rye, and wild rice [37]. While each of these is an excellent source of whole grains and related nutrients, they are infrequently consumed, particularly among children/adolescents. Arguably, whole-grain RTECs may provide an alternative way to increase whole grain consumption without compromising established preferences.

In general, replacing breakfast foods with RTECs led to higher quality diets. First, the contribution of whole grains to total grains increased from under 10% to 17.4% in Model 1 and 14.4% in Model 2. Even though these figures are below the recommended amount of 50%, they represent a substantial increase from the existing sub-optimal level. On the absolute scale, whole grains increased by 85% and 48% in Models 1 and 2, respectively, while refined grains were decreased non-significantly by 5.0% and 6.7%, respectively. Large increases were also observed for fiber, vitamin D, iron, folic acid, and magnesium. The effects on potassium were marginal, while total dairy and calcium increased by 11% and 15.8% in Model 2, respectively. While there was some evidence of an increase in added sugars (+5.5% in Model 1), this increase was offset by a decrease in solid fats (−10.9%), meaning that energy from added sugars and solid fats combined, marginally declined (−3.2%). Together, these components accounted for higher HEI-2010 scores. Importantly, these multiple dietary improvements were obtained without a corresponding increase in diet cost. In past studies, population-wide changes in HEI scores, and also some nutrients, were associated with higher diet costs [23,38,39]. However, making judicious food choices may allow some population groups to eat better for less.

Replacing breakfast foods with RTECs is just one of many strategies that can improve the overall diet quality. Other strategies may be less dependent on processed food. There has been some concern that the consumption of processed foods may have a deleterious impact on diet quality, particularly for consumption of dietary constituents to limit, including added sugars, some types of fat (mainly trans-fat), refined grains and sodium [40,41]. Replacing breakfast foods with whole fruit or low-fat yogurt would be consistent with food-based dietary guidelines, while providing important amounts of fiber, potassium and calcium, all nutrients of concern in the US. While some varieties of ready-to-eat cereal do contain added sugars, we previously found that they contribute 6.7% and 4.9% of added sugars among children 6–11 years and 12–19 years, respectively, compared to 37% and 55% of total added sugars from sugar-sweetened beverages [42]. Some have noted that processed foods fall along a continuum, some containing only empty calories, and others being more nutrient dense or containing food groups-to-encourage [43]. An analysis of 2003–2008 NHANES data showed that processed foods contributed 55% of dietary fiber, 48% of calcium and 43% of potassium, suggesting that on average, processed foods make an important contribution to the American diet, though they did provide as much as 57% of total energy [43]. Increasing the consumption of whole grains, an important public health priority, does require some processed foods, such as yeast breads, rolls, crackers, pasta or ready-to-eat cereals. Most grains in the US food supply, both refined and whole, are processed and are rarely cooked from scratch by the consumer, with the exception of brown rice and some other cooked grains [44]. This is the case for foods traditionally eaten at breakfast, with oatmeal being one notable exception. Compared to RTECs, oatmeal is eaten much less often [45].

The impact of the substitution models on a summary measure of adherence to the 2010 Dietary Guidelines for Americans, the HEI-2010, was also examined. For none of the models and age groups, did the HEI-2010 score decrease, indicating that on aggregate, the current models improved adherence to the DGAs. Future modeling work could examine other substitutions, including for other meals and eating occasions, but care should be made to ensure that the proposed dietary patterns are not only consistent with the DGAs, but also as cost-neutral as possible.

The limitations of this study are worth noting. First, this modeling study did not measure actual individual dietary behaviors, and rather evaluated the maximum effect of replacing breakfast foods with RTECs or RTECs and milk. While the model replaced many less healthful foods with RTECs (e.g., doughnuts, sweet rolls and pastries, and sausages and frankfurters provided 4.7% and 2.2% of breakfast calories, respectively, as shown in Supplemental Table S42), a number of healthful foods were also replaced (e.g., bananas and yogurt provided 1.8% and 1.0% of total breakfast calories, respectively). We did not assess the frequency with which sugar or other sweeteners were added to cereals, which may under-estimate, by a small amount, added sugar intake. In addition, we modeled RTEC cereals as they are currently consumed, which included both whole grain and non-whole grain cereals. If a shift towards consuming more whole grain or lower-sugar RTECs occurred, the positive results observed here would be stronger.

## 5. Conclusions

Substitution modeling, based on age and race specific criteria, in the context of a specific meal or eating occasion, offers one way to test the potential nutritional and economic impact of dietary guidance. The modeling of nationally representative dietary data can provide added insight into the impact of such policies on diet quality in the US [39,40]. Substitution modeling can be extended to evaluate, compare and rank the potential population-wide nutritional impact of proposed dietary changes. Such models may have practical use as tools for crafting effective nutrition communications.

## Figures and Tables

**Table 1:** Healthy Eating Index-2010 ^a^ values in observed diets and in replacement models using RTEC alone (Model 1) and RTEC plus milk (Model 2) Healthy Eating Index—2010.

	Observed		Model 1 (RTE)		Model 2 (RTE + Milk)	
	Mean	95% CI	Mean	95% CI	Mean	95% CI
*Population group*						
Total population (*n* = 18,112)	54.6	53.4, 55.9	56.0	54.9, 57.1	57.1 **	56.0, 58.2
Age 1–3 years (*n* = 1484)	56.3	55.0, 57.5	58.6 *	57.2, 60.1	57.8	56.4, 59.2
Age 4–8 years (*n* = 1895)	52.2	51.1, 53.3	52.8	51.6, 53.9	53.6	52.2, 54.9
Age 9–13 years (*n* = 1717)	47.4	45.5, 49.3	47.8	46.2, 49.5	49.6	48.0, 51.2
Age 14–19 years (*n* = 1834)	45.5	43.8, 47.2	46.3	44.5, 48.0	47.5	45.8, 49.3
Age 20–30 years (*n* = 1974)	49.2	47.5, 51.0	50.0	48.3, 51.6	51.1	49.5, 52.7
Age 31–50 years (*n* = 3.819)	54.1	52.0, 56.2	56.1	54.2, 58.0	57.1 *	55.2, 59.0
Age 51–70 years (*n* = 3513)	60.8	59.4, 62.1	63.3 **	62.0, 64.6	64.2 ***	62.9, 65.4
Age ≥71 years (*n* = 1876)	63.4	61.9, 64.9	65.5	64.0, 66.9	66.7 **	65.3, 68.2
*Special populations*						
WIC children (*n* = 1230)	56.3	55.0, 57.5	57.0	55.8, 58.2	56.4	55.2, 57.6

^a^ The HEI-2010 is a 100-point score ranging from 0 to 100, which measures adherence to the 2010 Dietary Guidelines for Americans. A score of 100 indicates perfect adherence to the 2010 Dietary Guidelines for Americans. Higher scores indicate higher quality diets. HEI-2010 is estimated using the population ratio method. Observed values serve as the reference group for statistical comparisons; *** *p* < 0.001; ** 0.001 < *p* < 0.01; * *p* < 0.05. WIC stands for Women, Infants and Children and indicates participation in this supplemental nutrition program. RTE: Ready-To-Eat.

## Data Availability

All data used in this research were publicly available as described below. The authors do not own the data. Questionnaires, Datasets, and Related Documentation. (http://www.cdc.gov/nchs/nhanes/nhanes_questionnaires.htm) [34]. Food Patterns Equivalents Database. (http://www.ars.usda.gov/Services/docs.htm?docid=23869) [46]. CNPP Food Price Database, 2003–2014. (http://www.cnpp.usda.gov/sites/default/files/usda_food_plans_cost_of_food/FoodPricesDatabase0304.XLS) [47].

## Supplementary Materials

The following are available online at www.mdpi.com/2072-6643/9/9/1010/s1, Tables S1–S41: Weighted ready-to-eat cereals by age and race/ethnicity group. RTECs contributing more than 1% weight are shown; Table S42: Sources of breakfast energy in the United States, 2007–2010.
