# Supplementary material for: Replacing American Breakfast Foods with Ready-To-Eat (RTE) Cereals Increases Consumption of Key Food Groups and Nutrients among US Children and Adults: Results of an NHANES Modeling Study
Source: Nutrients. 2017 Sep 13;9(9):1010. doi: 10.3390/nu9091010 (PMC5622770; doi:10.3390/nu9091010)
Supplement: Supplementary file 1 [file nutrients-09-01010-s001.zip › nutrients-220079-supplementary.pdf]

Supplemental Tables

Supplemental Tables S1–S41. Weighted ready-to-eat cereals by age and race/ethnicity group. RTECs contributing more than 1% weight are shown.

POPULATION	CEREAL NAME	WEIGHT
NON-HISPANIC WHITE, AGE 1-3	CHEERIOS	0.17
NON-HISPANIC WHITE, AGE 1-3	FROOT LOOPS CEREAL	0.06
NON-HISPANIC WHITE, AGE 1-3	HONEY NUT CHEERIOS	0.05
NON-HISPANIC WHITE, AGE 1-3	RICE KRISPIES, KELLOGG'S	0.05
NON-HISPANIC WHITE, AGE 1-3	LUCKY CHARMS CEREAL	0.05
NON-HISPANIC WHITE, AGE 1-3	FRUITY PEBBLES CEREAL	0.04
NON-HISPANIC WHITE, AGE 1-3	FROSTED MINI-WHEATS CEREAL (INCL ALL FLAVORS)	0.04
NON-HISPANIC WHITE, AGE 1-3	CAP'N CRUNCH CEREAL	0.03
NON-HISPANIC WHITE, AGE 1-3	GRANOLA, NFS	0.02
NON-HISPANIC WHITE, AGE 1-3	KIX CEREAL	0.02
NON-HISPANIC WHITE, AGE 1-3	CEREAL, READY-TO-EAT, NFS	0.02
NON-HISPANIC WHITE, AGE 1-3	FROSTED FLAKES, KELLOGG'S	0.02
NON-HISPANIC WHITE, AGE 1-3	CINNAMON TOAST CRUNCH CEREAL	0.02
NON-HISPANIC WHITE, AGE 1-3	SHREDDED WHEAT, 100%	0.02
NON-HISPANIC WHITE, AGE 1-3	LIFE CEREAL (PLAIN & CINNAMON)	0.02
NON-HISPANIC WHITE, AGE 1-3	RICE CHEX CEREAL	0.02
NON-HISPANIC WHITE, AGE 1-3	APPLE JACKS CEREAL	0.02
NON-HISPANIC WHITE, AGE 1-3	COCOA PUFFS CEREAL	0.02
NON-HISPANIC WHITE, AGE 1-3	TRIX CEREAL	0.01
NON-HISPANIC WHITE, AGE 1-3	CORN FLAKES, KELLOGG'S	0.01
NON-HISPANIC WHITE, AGE 1-3	OTHER	0.27

POPULATION	CEREAL NAME	WEIGHT
NON-HISPANIC BLACK, AGE 1-3	FROOT LOOPS CEREAL	0.12
NON-HISPANIC BLACK, AGE 1-3	CHEERIOS	0.11
NON-HISPANIC BLACK, AGE 1-3	HONEY NUT CHEERIOS	0.09
NON-HISPANIC BLACK, AGE 1-3	FROSTED FLAKES, KELLOGG'S	0.08
NON-HISPANIC BLACK, AGE 1-3	FRUITY PEBBLES CEREAL	0.07
NON-HISPANIC BLACK, AGE 1-3	CINNAMON TOAST CRUNCH CEREAL	0.06
NON-HISPANIC BLACK, AGE 1-3	CORN FLAKES, KELLOGG'S	0.05
NON-HISPANIC BLACK, AGE 1-3	RICE KRISPIES, KELLOGG'S	0.04
NON-HISPANIC BLACK, AGE 1-3	LUCKY CHARMS CEREAL	0.03
NON-HISPANIC BLACK, AGE 1-3	HONEY BUNCHES OF OATS CEREAL	0.03
NON-HISPANIC BLACK, AGE 1-3	CAP'N CRUNCH CEREAL	0.03
NON-HISPANIC BLACK, AGE 1-3	APPLE JACKS CEREAL	0.02
NON-HISPANIC BLACK, AGE 1-3	FROSTED MINI-WHEATS CEREAL (INCL ALL FLAVORS)	0.02
NON-HISPANIC BLACK, AGE 1-3	KIX CEREAL	0.02
NON-HISPANIC BLACK, AGE 1-3	REESE'S PEANUT BUTTER PUFFS CEREAL	0.02

NON-HISPANIC BLACK, AGE 1-3	TRIX CEREAL	0.02
NON-HISPANIC BLACK, AGE 1-3	CORN POPS CEREAL	0.02
NON-HISPANIC BLACK, AGE 1-3	MULTIGRAIN CHEERIOS	0.01
NON-HISPANIC BLACK, AGE 1-3	APPLE CINNAMON CHEERIOS	0.01
NON-HISPANIC BLACK, AGE 1-3	CRISP CRUNCH CEREAL	0.01
NON-HISPANIC BLACK, AGE 1-3	OTHER	0.16

POPULATION	CEREAL NAME	WEIGHT
MEXICAN-AMERICAN, AGE 1-3	CHEERIOS	0.13
MEXICAN-AMERICAN, AGE 1-3	HONEY NUT CHEERIOS	0.10
MEXICAN-AMERICAN, AGE 1-3	CORN FLAKES, KELLOGG'S	0.07
MEXICAN-AMERICAN, AGE 1-3	HONEY BUNCHES OF OATS CEREAL	0.07
MEXICAN-AMERICAN, AGE 1-3	LUCKY CHARMS CEREAL	0.06
MEXICAN-AMERICAN, AGE 1-3	FROOT LOOPS CEREAL	0.05
MEXICAN-AMERICAN, AGE 1-3	CORN FLAKES, NFS (INCLUDE STORE BRANDS)	0.04
MEXICAN-AMERICAN, AGE 1-3	FROSTED FLAKES, KELLOGG'S	0.04
MEXICAN-AMERICAN, AGE 1-3	HONEY BUNCHES OF OATS W/ ALMONDS, POST	0.04
MEXICAN-AMERICAN, AGE 1-3	CEREAL, READY-TO-EAT, NFS	0.03
MEXICAN-AMERICAN, AGE 1-3	KIX CEREAL	0.03
MEXICAN-AMERICAN, AGE 1-3	FRUITY PEBBLES CEREAL	0.03
MEXICAN-AMERICAN, AGE 1-3	COCOA KRISPIES CEREAL	0.02
MEXICAN-AMERICAN, AGE 1-3	CINNAMON TOAST CRUNCH CEREAL	0.02
MEXICAN-AMERICAN, AGE 1-3	DORA THE EXPLORER CEREAL	0.02
MEXICAN-AMERICAN, AGE 1-3	COCOA PUFFS CEREAL	0.02
MEXICAN-AMERICAN, AGE 1-3	RICE KRISPIES, KELLOGG'S	0.02
MEXICAN-AMERICAN, AGE 1-3	RAISIN BRAN, KELLOGG'S	0.02
MEXICAN-AMERICAN, AGE 1-3	TRIX CEREAL	0.01
MEXICAN-AMERICAN, AGE 1-3	MULTIGRAIN CHEERIOS	0.01
MEXICAN-AMERICAN, AGE 1-3	OTHER	0.16

POPULATION	CEREAL NAME	WEIGHT
OTHER HISPANIC, AGE 1-3	CHEERIOS	0.14
OTHER HISPANIC, AGE 1-3	FROOT LOOPS CEREAL	0.09
OTHER HISPANIC, AGE 1-3	CORN FLAKES, KELLOGG'S	0.09
OTHER HISPANIC, AGE 1-3	LUCKY CHARMS CEREAL	0.06
OTHER HISPANIC, AGE 1-3	HONEY NUT CHEERIOS	0.05
OTHER HISPANIC, AGE 1-3	HONEY BUNCHES OF OATS CEREAL	0.05
OTHER HISPANIC, AGE 1-3	CORN POPS CEREAL	0.05
OTHER HISPANIC, AGE 1-3	FRUITY PEBBLES CEREAL	0.04
OTHER HISPANIC, AGE 1-3	COCOA PUFFS CEREAL	0.04
OTHER HISPANIC, AGE 1-3	FROSTED FLAKES, KELLOGG'S	0.03
OTHER HISPANIC, AGE 1-3	REESE'S PEANUT BUTTER PUFFS CEREAL	0.03
OTHER HISPANIC, AGE 1-3	CAP'N CRUNCH'S CRUNCH BERRIES CEREAL	0.03
OTHER HISPANIC, AGE 1-3	RICE KRISPIES, KELLOGG'S	0.02

OTHER HISPANIC, AGE 1-3	GRANOLA, NFS	0.02
OTHER HISPANIC, AGE 1-3	CEREAL, READY-TO-EAT, NFS	0.02
OTHER HISPANIC, AGE 1-3	KIX CEREAL	0.02
OTHER HISPANIC, AGE 1-3	COCOA KRISPIES CEREAL	0.02
OTHER HISPANIC, AGE 1-3	BANANA NUT CRUNCH CEREAL (POST)	0.02
OTHER HISPANIC, AGE 1-3	CAP'N CRUNCH CEREAL	0.02
OTHER HISPANIC, AGE 1-3	MALT-O-MEAL MARSHMALLOW MATEYS CEREAL	0.02
OTHER HISPANIC, AGE 1-3	OTHER	0.15

POPULATION	CEREAL NAME	WEIGHT
OTHER/MIXED RACE, AGE 1-3	CHEERIOS	0.15
OTHER/MIXED RACE, AGE 1-3	FROOT LOOPS CEREAL	0.11
OTHER/MIXED RACE, AGE 1-3	HONEY NUT CHEERIOS	0.10
OTHER/MIXED RACE, AGE 1-3	HONEY BUNCHES OF OATS W/ ALMONDS, POST	0.10
OTHER/MIXED RACE, AGE 1-3	LIFE CEREAL (PLAIN & CINNAMON)	0.09
OTHER/MIXED RACE, AGE 1-3	LUCKY CHARMS CEREAL	0.07
OTHER/MIXED RACE, AGE 1-3	COCOA PUFFS CEREAL	0.04
OTHER/MIXED RACE, AGE 1-3	HONEY BUNCHES OF OATS CEREAL	0.03
OTHER/MIXED RACE, AGE 1-3	HONEYCOMB CEREAL, PLAIN	0.03
OTHER/MIXED RACE, AGE 1-3	MULTIGRAIN CHEERIOS	0.03
OTHER/MIXED RACE, AGE 1-3	BANANA NUT CRUNCH CEREAL (POST)	0.02
OTHER/MIXED RACE, AGE 1-3	TRIX CEREAL	0.02
OTHER/MIXED RACE, AGE 1-3	CEREAL, READY-TO-EAT, NFS	0.02
OTHER/MIXED RACE, AGE 1-3	FROSTED MINI-WHEATS CEREAL (INCL ALL FLAVORS)	0.02
OTHER/MIXED RACE, AGE 1-3	CORN FLAKES, KELLOGG'S	0.02
OTHER/MIXED RACE, AGE 1-3	CINNAMON TOAST CRUNCH CEREAL	0.02
OTHER/MIXED RACE, AGE 1-3	RICE KRISPIES, KELLOGG'S	0.02
OTHER/MIXED RACE, AGE 1-3	FROSTED FLAKES, KELLOGG'S	0.01
OTHER/MIXED RACE, AGE 1-3	OH'S, HONEY GRAHAM CEREAL	0.01
OTHER/MIXED RACE, AGE 1-3	RAISIN BRAN, KELLOGG'S	0.01
OTHER/MIXED RACE, AGE 1-3	OTHER	0.08

POPULATION	CEREAL NAME	WEIGHT
NON-HISPANIC WHITE, AGE 4-8	LUCKY CHARMS CEREAL	0.07
NON-HISPANIC WHITE, AGE 4-8	FROOT LOOPS CEREAL	0.07
NON-HISPANIC WHITE, AGE 4-8	HONEY NUT CHEERIOS	0.05
NON-HISPANIC WHITE, AGE 4-8	FROSTED MINI-WHEATS CEREAL (INCL ALL FLAVORS)	0.04
NON-HISPANIC WHITE, AGE 4-8	FRUITY PEBBLES CEREAL	0.04
NON-HISPANIC WHITE, AGE 4-8	CINNAMON TOAST CRUNCH CEREAL	0.04
NON-HISPANIC WHITE, AGE 4-8	FROSTED FLAKES, KELLOGG'S	0.04
NON-HISPANIC WHITE, AGE 4-8	RICE KRISPIES, KELLOGG'S	0.04
NON-HISPANIC WHITE, AGE 4-8	REESE'S PEANUT BUTTER PUFFS CEREAL	0.04
NON-HISPANIC WHITE, AGE 4-8	COCOA PUFFS CEREAL	0.03
NON-HISPANIC WHITE, AGE 4-8	CAP'N CRUNCH'S PEANUT BUTTER CRUNCH CEREAL	0.03

NON-HISPANIC WHITE, AGE 4-8	CEREAL, READY-TO-EAT, NFS	0.03
NON-HISPANIC WHITE, AGE 4-8	COCOA KRISPIES CEREAL	0.03
NON-HISPANIC WHITE, AGE 4-8	FROSTED WHEAT BITES	0.02
NON-HISPANIC WHITE, AGE 4-8	CAP'N CRUNCH'S CRUNCH BERRIES CEREAL	0.02
NON-HISPANIC WHITE, AGE 4-8	CHEERIOS	0.02
NON-HISPANIC WHITE, AGE 4-8	LIFE CEREAL (PLAIN & CINNAMON)	0.02
NON-HISPANIC WHITE, AGE 4-8	COCOA PEBBLES CEREAL	0.02
NON-HISPANIC WHITE, AGE 4-8	COOKIE-CRISP CEREAL (INCLUDE ALL FLAVORS)	0.02
NON-HISPANIC WHITE, AGE 4-8	CORN POPS CEREAL	0.02
NON-HISPANIC WHITE, AGE 4-8	OTHER	0.32

POPULATION	CEREAL NAME	WEIGHT
NON-HISPANIC BLACK, AGE 4-8	FROSTED FLAKES, KELLOGG'S	0.12
NON-HISPANIC BLACK, AGE 4-8	FROOT LOOPS CEREAL	0.10
NON-HISPANIC BLACK, AGE 4-8	HONEY NUT CHEERIOS	0.08
NON-HISPANIC BLACK, AGE 4-8	CINNAMON TOAST CRUNCH CEREAL	0.08
NON-HISPANIC BLACK, AGE 4-8	LUCKY CHARMS CEREAL	0.04
NON-HISPANIC BLACK, AGE 4-8	FRUITY PEBBLES CEREAL	0.04
NON-HISPANIC BLACK, AGE 4-8	APPLE JACKS CEREAL	0.04
NON-HISPANIC BLACK, AGE 4-8	COCOA PUFFS CEREAL	0.03
NON-HISPANIC BLACK, AGE 4-8	RICE KRISPIES, KELLOGG'S	0.03
NON-HISPANIC BLACK, AGE 4-8	CHEERIOS	0.03
NON-HISPANIC BLACK, AGE 4-8	CORN FLAKES, KELLOGG'S	0.03
NON-HISPANIC BLACK, AGE 4-8	TRIX CEREAL	0.02
NON-HISPANIC BLACK, AGE 4-8	RAISIN BRAN, KELLOGG'S	0.02
NON-HISPANIC BLACK, AGE 4-8	GOLDEN GRAHAMS CEREAL	0.02
NON-HISPANIC BLACK, AGE 4-8	CAP'N CRUNCH CEREAL	0.02
NON-HISPANIC BLACK, AGE 4-8	HONEYCOMB CEREAL, PLAIN	0.02
NON-HISPANIC BLACK, AGE 4-8	CAP'N CRUNCH'S CRUNCH BERRIES CEREAL	0.02
NON-HISPANIC BLACK, AGE 4-8	HONEY BUNCHES OF OATS CEREAL	0.02
NON-HISPANIC BLACK, AGE 4-8	COCOA KRISPIES CEREAL	0.02
NON-HISPANIC BLACK, AGE 4-8	CEREAL, READY-TO-EAT, NFS	0.02
NON-HISPANIC BLACK, AGE 4-8	OTHER	0.19

POPULATION	CEREAL NAME	WEIGHT
MEXICAN-AMERICAN, AGE 4-8	HONEY NUT CHEERIOS	0.11
MEXICAN-AMERICAN, AGE 4-8	CORN FLAKES, KELLOGG'S	0.09
MEXICAN-AMERICAN, AGE 4-8	FROOT LOOPS CEREAL	0.06
MEXICAN-AMERICAN, AGE 4-8	FROSTED FLAKES, KELLOGG'S	0.06
MEXICAN-AMERICAN, AGE 4-8	CHEERIOS	0.05
MEXICAN-AMERICAN, AGE 4-8	LUCKY CHARMS CEREAL	0.05
MEXICAN-AMERICAN, AGE 4-8	HONEY BUNCHES OF OATS CEREAL	0.04
MEXICAN-AMERICAN, AGE 4-8	CEREAL, READY-TO-EAT, NFS	0.04
MEXICAN-AMERICAN, AGE 4-8	HONEY BUNCHES OF OATS W/ ALMONDS, POST	0.04

MEXICAN-AMERICAN, AGE 4-8	FRUITY PEBBLES CEREAL	0.04
MEXICAN-AMERICAN, AGE 4-8	CINNAMON TOAST CRUNCH CEREAL	0.03
MEXICAN-AMERICAN, AGE 4-8	COCOA PUFFS CEREAL	0.03
MEXICAN-AMERICAN, AGE 4-8	APPLE JACKS CEREAL	0.03
MEXICAN-AMERICAN, AGE 4-8	COCOA KRISPIES CEREAL	0.03
MEXICAN-AMERICAN, AGE 4-8	FROSTED MINI-WHEATS CEREAL (INCL ALL FLAVORS)	0.02
MEXICAN-AMERICAN, AGE 4-8	CORN FLAKES, NFS (INCLUDE STORE BRANDS)	0.02
MEXICAN-AMERICAN, AGE 4-8	CAP'N CRUNCH CEREAL	0.02
MEXICAN-AMERICAN, AGE 4-8	KIX CEREAL	0.02
MEXICAN-AMERICAN, AGE 4-8	RICE KRISPIES, KELLOGG'S	0.02
MEXICAN-AMERICAN, AGE 4-8	TRIX CEREAL	0.01
MEXICAN-AMERICAN AGE 4-8	OTHER	0.19

POPULATION	CEREAL NAME	WEIGHT
OTHER HISPANIC, AGE 4-8	FROSTED FLAKES, KELLOGG'S	0.13
OTHER HISPANIC, AGE 4-8	HONEY NUT CHEERIOS	0.07
OTHER HISPANIC, AGE 4-8	CHEERIOS	0.07
OTHER HISPANIC, AGE 4-8	FROOT LOOPS CEREAL	0.06
OTHER HISPANIC, AGE 4-8	CINNAMON TOAST CRUNCH CEREAL	0.06
OTHER HISPANIC, AGE 4-8	CORN FLAKES, KELLOGG'S	0.06
OTHER HISPANIC, AGE 4-8	CEREAL, READY-TO-EAT, NFS	0.05
OTHER HISPANIC, AGE 4-8	HONEY BUNCHES OF OATS CEREAL	0.05
OTHER HISPANIC, AGE 4-8	COCOA PUFFS CEREAL	0.04
OTHER HISPANIC, AGE 4-8	COCOA PEBBLES CEREAL	0.03
OTHER HISPANIC, AGE 4-8	LUCKY CHARMS CEREAL	0.03
OTHER HISPANIC, AGE 4-8	FRUITY PEBBLES CEREAL	0.03
OTHER HISPANIC, AGE 4-8	TRIX CEREAL	0.03
OTHER HISPANIC, AGE 4-8	COCOA KRISPIES CEREAL	0.03
OTHER HISPANIC, AGE 4-8	KIX CEREAL	0.02
OTHER HISPANIC, AGE 4-8	CORN POPS CEREAL	0.02
OTHER HISPANIC, AGE 4-8	MALT-O-MEAL MARSHMALLOW MATEYS CEREAL	0.01
OTHER HISPANIC, AGE 4-8	HONEY SMACKS, KELLOGG'S	0.01
OTHER HISPANIC, AGE 4-8	SPECIAL K VANILLA ALMOND	0.01
OTHER HISPANIC, AGE 4-8	BANANA NUT CRUNCH CEREAL (POST)	0.01
OTHER HISPANIC, AGE 4-8	OTHER	0.17

POPULATION	CEREAL NAME	WEIGHT
OTHER/MIXED RACE, AGE 4-8	HONEY NUT CHEERIOS	0.11
OTHER/MIXED RACE, AGE 4-8	FROOT LOOPS CEREAL	0.11
OTHER/MIXED RACE, AGE 4-8	LUCKY CHARMS CEREAL	0.08
OTHER/MIXED RACE, AGE 4-8	CINNAMON TOAST CRUNCH CEREAL	0.08
OTHER/MIXED RACE, AGE 4-8	FROSTED FLAKES, KELLOGG'S	0.07
OTHER/MIXED RACE, AGE 4-8	RICE KRISPIES, KELLOGG'S	0.07
OTHER/MIXED RACE, AGE 4-8	REESE'S PEANUT BUTTER PUFFS CEREAL	0.05

OTHER/MIXED RACE, AGE 4-8	COCOA PUFFS CEREAL	0.05
OTHER/MIXED RACE, AGE 4-8	FROSTED OAT CEREAL W/ MARSHMALLOWS	0.04
OTHER/MIXED RACE, AGE 4-8	HONEY BUNCHES OF OATS CEREAL	0.04
OTHER/MIXED RACE, AGE 4-8	GRANOLA, NFS	0.04
OTHER/MIXED RACE, AGE 4-8	COCOA KRISPIES CEREAL	0.03
OTHER/MIXED RACE, AGE 4-8	HONEY BUNCHES OF OATS W/ ALMONDS, POST	0.03
OTHER/MIXED RACE, AGE 4-8	TRIX CEREAL	0.03
OTHER/MIXED RACE, AGE 4-8	100% NATURAL CEREAL, PLAIN, QUAKER	0.02
OTHER/MIXED RACE, AGE 4-8	CHEERIOS	0.02
OTHER/MIXED RACE, AGE 4-8	SPECIAL K RED BERRIES	0.02
OTHER/MIXED RACE, AGE 4-8	APPLE JACKS CEREAL	0.02
OTHER/MIXED RACE, AGE 4-8	COOKIE-CRISP CEREAL (INCLUDE ALL FLAVORS)	0.02
OTHER/MIXED RACE, AGE 4-8	FRUITY PEBBLES CEREAL	0.02
OTHER/MIXED RACE, AGE 4-8	OTHER	0.08

POPULATION	CEREAL NAME	WEIGHT
NON-HISPANIC WHITE, AGE 9-13	CINNAMON TOAST CRUNCH CEREAL	0.06
NON-HISPANIC WHITE, AGE 9-13	HONEY NUT CHEERIOS	0.05
NON-HISPANIC WHITE, AGE 9-13	LUCKY CHARMS CEREAL	0.05
NON-HISPANIC WHITE, AGE 9-13	COCOA PUFFS CEREAL	0.05
NON-HISPANIC WHITE, AGE 9-13	FRUITY PEBBLES CEREAL	0.05
NON-HISPANIC WHITE, AGE 9-13	COCOA PEBBLES CEREAL	0.04
NON-HISPANIC WHITE, AGE 9-13	FROSTED FLAKES, KELLOGG'S	0.04
NON-HISPANIC WHITE, AGE 9-13	CAP'N CRUNCH CEREAL	0.04
NON-HISPANIC WHITE, AGE 9-13	FROSTED MINI-WHEATS CEREAL (INCL ALL FLAVORS)	0.03
NON-HISPANIC WHITE, AGE 9-13	APPLE JACKS CEREAL	0.03
NON-HISPANIC WHITE, AGE 9-13	REESE'S PEANUT BUTTER PUFFS CEREAL	0.03
NON-HISPANIC WHITE, AGE 9-13	RICE KRISPIES, KELLOGG'S	0.03
NON-HISPANIC WHITE, AGE 9-13	HONEY BUNCHES OF OATS CEREAL	0.03
NON-HISPANIC WHITE, AGE 9-13	CHEERIOS	0.03
NON-HISPANIC WHITE, AGE 9-13	FROOT LOOPS CEREAL	0.03
NON-HISPANIC WHITE, AGE 9-13	COOKIE-CRISP CEREAL (INCLUDE ALL FLAVORS)	0.03
NON-HISPANIC WHITE, AGE 9-13	FROSTED CORN FLAKES, NFS	0.02
NON-HISPANIC WHITE, AGE 9-13	HONEY NUT CLUSTERS CEREAL	0.02
NON-HISPANIC WHITE, AGE 9-13	LIFE CEREAL (PLAIN & CINNAMON)	0.02
NON-HISPANIC WHITE, AGE 9-13	SPECIAL K CEREAL	0.02
NON-HISPANIC WHITE, AGE 9-13	OTHER	0.31

POPULATION	CEREAL NAME	WEIGHT
NON-HISPANIC BLACK, AGE 9-13	FROSTED FLAKES, KELLOGG'S	0.15
NON-HISPANIC BLACK, AGE 9-13	FROOT LOOPS CEREAL	0.10
NON-HISPANIC BLACK, AGE 9-13	CINNAMON TOAST CRUNCH CEREAL	0.07
NON-HISPANIC BLACK, AGE 9-13	CAP'N CRUNCH'S CRUNCH BERRIES CEREAL	0.05
NON-HISPANIC BLACK, AGE 9-13	APPLE JACKS CEREAL	0.05

NON-HISPANIC BLACK, AGE 9-13	LUCKY CHARMS CEREAL	0.05
NON-HISPANIC BLACK, AGE 9-13	FRUITY PEBBLES CEREAL	0.04
NON-HISPANIC BLACK, AGE 9-13	RAISIN BRAN, KELLOGG'S	0.04
NON-HISPANIC BLACK, AGE 9-13	CHEERIOS	0.03
NON-HISPANIC BLACK, AGE 9-13	CAP'N CRUNCH CEREAL	0.03
NON-HISPANIC BLACK, AGE 9-13	REESE'S PEANUT BUTTER PUFFS CEREAL	0.03
NON-HISPANIC BLACK, AGE 9-13	HONEY BUNCHES OF OATS CEREAL	0.03
NON-HISPANIC BLACK, AGE 9-13	HONEY NUT CHEERIOS	0.03
NON-HISPANIC BLACK, AGE 9-13	FROSTED MINI-WHEATS CEREAL (INCL ALL FLAVORS)	0.03
NON-HISPANIC BLACK, AGE 9-13	COCOA PUFFS CEREAL	0.03
NON-HISPANIC BLACK, AGE 9-13	HONEY SMACKS, KELLOGG'S	0.02
NON-HISPANIC BLACK, AGE 9-13	FROSTED CORN FLAKES, NFS	0.01
NON-HISPANIC BLACK, AGE 9-13	CORN FLAKES, KELLOGG'S	0.01
NON-HISPANIC BLACK, AGE 9-13	FRENCH TOAST CRUNCH CEREAL, GENERAL MILLS	0.01
NON-HISPANIC BLACK, AGE 9-13	COOKIE-CRISP CEREAL (INCLUDE ALL FLAVORS)	0.01
NON-HISPANIC BLACK, AGE 9-13	OTHER	0.31

POPULATION	CEREAL NAME	WEIGHT
MEXICAN-AMERICAN, AGE 9-13	HONEY NUT CHEERIOS	0.09
MEXICAN-AMERICAN, AGE 9-13	HONEY BUNCHES OF OATS CEREAL	0.07
MEXICAN-AMERICAN, AGE 9-13	CHEERIOS	0.07
MEXICAN-AMERICAN, AGE 9-13	FROSTED FLAKES, KELLOGG'S	0.07
MEXICAN-AMERICAN, AGE 9-13	CORN FLAKES, KELLOGG'S	0.06
MEXICAN-AMERICAN, AGE 9-13	COCOA PUFFS CEREAL	0.06
MEXICAN-AMERICAN, AGE 9-13	FROOT LOOPS CEREAL	0.05
MEXICAN-AMERICAN, AGE 9-13	HONEY BUNCHES OF OATS W/ ALMONDS, POST	0.04
MEXICAN-AMERICAN, AGE 9-13	COCOA KRISPIES CEREAL	0.04
MEXICAN-AMERICAN, AGE 9-13	LUCKY CHARMS CEREAL	0.04
MEXICAN-AMERICAN, AGE 9-13	FRUITY PEBBLES CEREAL	0.04
MEXICAN-AMERICAN, AGE 9-13	FROSTED MINI-WHEATS CEREAL (INCL ALL FLAVORS)	0.03
MEXICAN-AMERICAN, AGE 9-13	CEREAL, READY-TO-EAT, NFS	0.02
MEXICAN-AMERICAN, AGE 9-13	CINNAMON TOAST CRUNCH CEREAL	0.02
MEXICAN-AMERICAN, AGE 9-13	TRIX CEREAL	0.02
MEXICAN-AMERICAN, AGE 9-13	CORN POPS CEREAL	0.02
MEXICAN-AMERICAN, AGE 9-13	LIFE CEREAL (PLAIN & CINNAMON)	0.02
MEXICAN-AMERICAN, AGE 9-13	CORN FLAKES, NFS (INCLUDE STORE BRANDS)	0.02
MEXICAN-AMERICAN, AGE 9-13	APPLE JACKS CEREAL	0.02
MEXICAN-AMERICAN, AGE 9-13	COOKIE-CRISP CEREAL (INCLUDE ALL FLAVORS)	0.02
MEXICAN-AMERICAN AGE 9-13	OTHER	0.31

POPULATION	CEREAL NAME	WEIGHT
OTHER HISPANIC, AGE 9-13	FROOT LOOPS CEREAL	0.10
OTHER HISPANIC, AGE 9-13	HONEY NUT CHEERIOS	0.08
OTHER HISPANIC, AGE 9-13	FROSTED FLAKES, KELLOGG'S	0.08

OTHER HISPANIC, AGE 9-13	FRUITY PEBBLES CEREAL	0.08
OTHER HISPANIC, AGE 9-13	LUCKY CHARMS CEREAL	0.08
OTHER HISPANIC, AGE 9-13	CORN FLAKES, KELLOGG'S	0.07
OTHER HISPANIC, AGE 9-13	HONEY BUNCHES OF OATS CEREAL	0.04
OTHER HISPANIC, AGE 9-13	CEREAL, READY-TO-EAT, NFS	0.04
OTHER HISPANIC, AGE 9-13	FROSTED MINI-WHEATS CEREAL (INCL ALL FLAVORS)	0.03
OTHER HISPANIC, AGE 9-13	CHEERIOS	0.03
OTHER HISPANIC, AGE 9-13	RICE KRISPIES, KELLOGG'S	0.03
OTHER HISPANIC, AGE 9-13	REESE'S PEANUT BUTTER PUFFS CEREAL	0.02
OTHER HISPANIC, AGE 9-13	CAP'N CRUNCH'S CRUNCH BERRIES CEREAL	0.02
OTHER HISPANIC, AGE 9-13	CINNAMON TOAST CRUNCH CEREAL	0.02
OTHER HISPANIC, AGE 9-13	CAP'N CRUNCH CEREAL	0.02
OTHER HISPANIC, AGE 9-13	APPLE JACKS CEREAL	0.02
OTHER HISPANIC, AGE 9-13	FROSTED CHEERIOS CEREAL	0.02
OTHER HISPANIC, AGE 9-13	COCOA PUFFS CEREAL	0.02
OTHER HISPANIC, AGE 9-13	GOLDEN GRAHAMS CEREAL	0.02
OTHER HISPANIC, AGE 9-13	COOKIE-CRISP CEREAL (INCLUDE ALL FLAVORS)	0.01
OTHER HISPANIC, AGE 9-13	OTHER	0.31

POPULATION	CEREAL NAME	WEIGHT
OTHER/MIXED RACE, AGE 9-13	HONEY NUT CHEERIOS	0.10
OTHER/MIXED RACE, AGE 9-13	FRUITY PEBBLES CEREAL	0.08
OTHER/MIXED RACE, AGE 9-13	FROOT LOOPS CEREAL	0.07
OTHER/MIXED RACE, AGE 9-13	FROSTED MINI-WHEATS CEREAL (INCL ALL FLAVORS)	0.06
OTHER/MIXED RACE, AGE 9-13	CINNAMON TOAST CRUNCH CEREAL	0.06
OTHER/MIXED RACE, AGE 9-13	HONEY BUNCHES OF OATS CEREAL	0.06
OTHER/MIXED RACE, AGE 9-13	FROSTED FLAKES, KELLOGG'S	0.06
OTHER/MIXED RACE, AGE 9-13	CHEX CEREAL, NFS	0.05
OTHER/MIXED RACE, AGE 9-13	LUCKY CHARMS CEREAL	0.04
OTHER/MIXED RACE, AGE 9-13	REESE'S PEANUT BUTTER PUFFS CEREAL	0.04
OTHER/MIXED RACE, AGE 9-13	SHREDDED WHEAT, 100%	0.03
OTHER/MIXED RACE, AGE 9-13	CAP'N CRUNCH'S CRUNCH BERRIES CEREAL	0.03
OTHER/MIXED RACE, AGE 9-13	CORN FLAKES, KELLOGG'S	0.03
OTHER/MIXED RACE, AGE 9-13	KIX CEREAL	0.02
OTHER/MIXED RACE, AGE 9-13	MALT-O-MEAL MARSHMALLOW MATEYS CEREAL	0.02
OTHER/MIXED RACE, AGE 9-13	CORN CHEX CEREAL	0.02
OTHER/MIXED RACE, AGE 9-13	MINI-SWIRLZ CINNAMON BUN CEREAL, KELLOGG'S	0.02
OTHER/MIXED RACE, AGE 9-13	COOKIE-CRISP CEREAL (INCLUDE ALL FLAVORS)	0.02
OTHER/MIXED RACE, AGE 9-13	HONEY SMACKS, KELLOGG'S	0.02
OTHER/MIXED RACE, AGE 9-13	COCOA PUFFS CEREAL	0.02
OTHER/MIXED RACE, AGE 9-13	OTHER	0.31

POPULATION	CEREAL NAME	WEIGHT
NON-HISPANIC WHITE, AGE 14-19	FROSTED MINI-WHEATS CEREAL (INCL ALL FLAVORS)	0.08

NON-HISPANIC WHITE, AGE 14-19	FROSTED FLAKES, KELLOGG'S	0.07
NON-HISPANIC WHITE, AGE 14-19	LUCKY CHARMS CEREAL	0.07
NON-HISPANIC WHITE, AGE 14-19	FROOT LOOPS CEREAL	0.05
NON-HISPANIC WHITE, AGE 14-19	HONEY NUT CHEERIOS	0.05
NON-HISPANIC WHITE, AGE 14-19	CINNAMON TOAST CRUNCH CEREAL	0.04
NON-HISPANIC WHITE, AGE 14-19	FROSTED CORN FLAKES, NFS	0.04
NON-HISPANIC WHITE, AGE 14-19	REESE'S PEANUT BUTTER PUFFS CEREAL	0.03
NON-HISPANIC WHITE, AGE 14-19	FRUITY PEBBLES CEREAL	0.03
NON-HISPANIC WHITE, AGE 14-19	HONEY BUNCHES OF OATS W/ ALMONDS, POST	0.03
NON-HISPANIC WHITE, AGE 14-19	COCOA PUFFS CEREAL	0.03
NON-HISPANIC WHITE, AGE 14-19	HONEY BUNCHES OF OATS CEREAL	0.03
NON-HISPANIC WHITE, AGE 14-19	CHEERIOS	0.02
NON-HISPANIC WHITE, AGE 14-19	RICE KRISPIES, KELLOGG'S	0.02
NON-HISPANIC WHITE, AGE 14-19	LIFE CEREAL (PLAIN & CINNAMON)	0.02
NON-HISPANIC WHITE, AGE 14-19	RAISIN BRAN CEREAL, POST	0.02
NON-HISPANIC WHITE, AGE 14-19	CAP'N CRUNCH CEREAL	0.02
NON-HISPANIC WHITE, AGE 14-19	GRAPE-NUTS CEREAL	0.02
NON-HISPANIC WHITE, AGE 14-19	GRANOLA, NFS	0.02
NON-HISPANIC WHITE, AGE 14-19	APPLE JACKS CEREAL	0.02
NON-HISPANIC WHITE, AGE 14-19	OTHER	0.29

POPULATION	CEREAL NAME	WEIGHT
NON-HISPANIC BLACK, AGE 14-19	FROSTED FLAKES, KELLOGG'S	0.12
NON-HISPANIC BLACK, AGE 14-19	CINNAMON TOAST CRUNCH CEREAL	0.10
NON-HISPANIC BLACK, AGE 14-19	FROOT LOOPS CEREAL	0.10
NON-HISPANIC BLACK, AGE 14-19	CAP'N CRUNCH CEREAL	0.06
NON-HISPANIC BLACK, AGE 14-19	HONEY NUT CHEERIOS	0.06
NON-HISPANIC BLACK, AGE 14-19	FRUITY PEBBLES CEREAL	0.05
NON-HISPANIC BLACK, AGE 14-19	RAISIN BRAN, KELLOGG'S	0.04
NON-HISPANIC BLACK, AGE 14-19	APPLE JACKS CEREAL	0.04
NON-HISPANIC BLACK, AGE 14-19	CAP'N CRUNCH'S CRUNCH BERRIES CEREAL	0.04
NON-HISPANIC BLACK, AGE 14-19	COOKIE-CRISP CEREAL (INCLUDE ALL FLAVORS)	0.04
NON-HISPANIC BLACK, AGE 14-19	COCOA PUFFS CEREAL	0.03
NON-HISPANIC BLACK, AGE 14-19	COCOA PEBBLES CEREAL	0.03
NON-HISPANIC BLACK, AGE 14-19	FROSTED MINI-WHEATS CEREAL (INCL ALL FLAVORS)	0.03
NON-HISPANIC BLACK, AGE 14-19	TRIX CEREAL	0.02
NON-HISPANIC BLACK, AGE 14-19	CORN POPS CEREAL	0.02
NON-HISPANIC BLACK, AGE 14-19	REESE'S PEANUT BUTTER PUFFS CEREAL	0.02
NON-HISPANIC BLACK, AGE 14-19	RAISIN BRAN CEREAL, NFS	0.01
NON-HISPANIC BLACK, AGE 14-19	CORN FLAKES, KELLOGG'S	0.01
NON-HISPANIC BLACK, AGE 14-19	COCOA KRISPIES CEREAL	0.01
NON-HISPANIC BLACK, AGE 14-19	BERRY BURST CHEERIOS	0.01
NON-HISPANIC BLACK, AGE 14-19	OTHER	0.14

POPULATION	CEREAL NAME	WEIGHT
MEXICAN-AMERICAN, AGE 14-19	FROSTED FLAKES, KELLOGG'S	0.10
MEXICAN-AMERICAN, AGE 14-19	HONEY NUT CHEERIOS	0.08
MEXICAN-AMERICAN, AGE 14-19	HONEY BUNCHES OF OATS CEREAL	0.08
MEXICAN-AMERICAN, AGE 14-19	HONEY BUNCHES OF OATS W/ ALMONDS, POST	0.05
MEXICAN-AMERICAN, AGE 14-19	COCOA KRISPIES CEREAL	0.05
MEXICAN-AMERICAN, AGE 14-19	CORN FLAKES, KELLOGG'S	0.05
MEXICAN-AMERICAN, AGE 14-19	FROSTED CORN FLAKES, NFS	0.05
MEXICAN-AMERICAN, AGE 14-19	LUCKY CHARMS CEREAL	0.04
MEXICAN-AMERICAN, AGE 14-19	FRUITY PEBBLES CEREAL	0.04
MEXICAN-AMERICAN, AGE 14-19	COCOA PUFFS CEREAL	0.04
MEXICAN-AMERICAN, AGE 14-19	CHEERIOS	0.04
MEXICAN-AMERICAN, AGE 14-19	FROOT LOOPS CEREAL	0.03
MEXICAN-AMERICAN, AGE 14-19	CEREAL, READY-TO-EAT, NFS	0.03
MEXICAN-AMERICAN, AGE 14-19	FROSTED MINI-WHEATS CEREAL (INCL ALL FLAVORS)	0.03
MEXICAN-AMERICAN, AGE 14-19	CINNAMON TOAST CRUNCH CEREAL	0.03
MEXICAN-AMERICAN, AGE 14-19	LIFE CEREAL (PLAIN & CINNAMON)	0.02
MEXICAN-AMERICAN, AGE 14-19	SPECIAL K VANILLA ALMOND	0.02
MEXICAN-AMERICAN, AGE 14-19	SPECIAL K RED BERRIES	0.02
MEXICAN-AMERICAN, AGE 14-19	APPLE JACKS CEREAL	0.02
MEXICAN-AMERICAN, AGE 14-19	CORN FLAKES, NFS (INCLUDE STORE BRANDS)	0.01
MEXICAN-AMERICAN, AGE 14-19	OTHER	0.16

POPULATION	CEREAL NAME	WEIGHT
OTHER HISPANIC, AGE 14-19	HONEY NUT CHEERIOS	0.07
OTHER HISPANIC, AGE 14-19	CORN FLAKES, KELLOGG'S	0.07
OTHER HISPANIC, AGE 14-19	FROSTED FLAKES, KELLOGG'S	0.06
OTHER HISPANIC, AGE 14-19	FROSTED MINI-WHEATS CEREAL (INCL ALL FLAVORS)	0.06
OTHER HISPANIC, AGE 14-19	CINNAMON TOAST CRUNCH CEREAL	0.06
OTHER HISPANIC, AGE 14-19	FROOT LOOPS CEREAL	0.05
OTHER HISPANIC, AGE 14-19	LUCKY CHARMS CEREAL	0.04
OTHER HISPANIC, AGE 14-19	COCOA PEBBLES CEREAL	0.04
OTHER HISPANIC, AGE 14-19	COCOA PUFFS CEREAL	0.04
OTHER HISPANIC, AGE 14-19	TRIX CEREAL	0.03
OTHER HISPANIC, AGE 14-19	FRUITY PEBBLES CEREAL	0.03
OTHER HISPANIC, AGE 14-19	HONEY BUNCHES OF OATS CEREAL	0.03
OTHER HISPANIC, AGE 14-19	CAP'N CRUNCH'S CRUNCH BERRIES CEREAL	0.03
OTHER HISPANIC, AGE 14-19	CORN POPS CEREAL	0.03
OTHER HISPANIC, AGE 14-19	HONEY BUNCHES OF OATS W/ ALMONDS, POST	0.03
OTHER HISPANIC, AGE 14-19	APPLE JACKS CEREAL	0.03
OTHER HISPANIC, AGE 14-19	CEREAL, READY-TO-EAT, NFS	0.03
OTHER HISPANIC, AGE 14-19	FRENCH TOAST CRUNCH CEREAL, GENERAL MILLS	0.02
OTHER HISPANIC, AGE 14-19	CORN FLAKES, NFS (INCLUDE STORE BRANDS)	0.02
OTHER HISPANIC, AGE 14-19	RAISIN BRAN CRUNCH, KELLOGG'S	0.02

OTHER HISPANIC, AGE 14-19	OTHER	0.21

POPULATION	CEREAL NAME	WEIGHT
OTHER/MIXED RACE, AGE 14-19	HONEY NUT CHEERIOS	0.15
OTHER/MIXED RACE, AGE 14-19	LUCKY CHARMS CEREAL	0.12
OTHER/MIXED RACE, AGE 14-19	CAP'N CRUNCH'S PEANUT BUTTER CRUNCH CEREAL	0.06
OTHER/MIXED RACE, AGE 14-19	RAISIN BRAN, KELLOGG'S	0.06
OTHER/MIXED RACE, AGE 14-19	HONEY BUNCHES OF OATS CEREAL	0.05
OTHER/MIXED RACE, AGE 14-19	MALT-O-MEAL TOOTIE FRUITIES (RTE CEREAL)	0.05
OTHER/MIXED RACE, AGE 14-19	REESE'S PEANUT BUTTER PUFFS CEREAL	0.05
OTHER/MIXED RACE, AGE 14-19	CHEX CEREAL, NFS	0.04
OTHER/MIXED RACE, AGE 14-19	HONEY BUNCHES OF OATS W/ ALMONDS, POST	0.04
OTHER/MIXED RACE, AGE 14-19	RICE KRISPIES, KELLOGG'S	0.04
OTHER/MIXED RACE, AGE 14-19	FROSTED FLAKES, KELLOGG'S	0.04
OTHER/MIXED RACE, AGE 14-19	FROSTED MINI-WHEATS CEREAL (INCL ALL FLAVORS)	0.03
OTHER/MIXED RACE, AGE 14-19	COCOA PUFFS CEREAL	0.03
OTHER/MIXED RACE, AGE 14-19	CEREAL, READY-TO-EAT, NFS	0.03
OTHER/MIXED RACE, AGE 14-19	RAISIN BRAN CEREAL, POST	0.02
OTHER/MIXED RACE, AGE 14-19	CHEERIOS	0.02
OTHER/MIXED RACE, AGE 14-19	FROSTED CHEERIOS CEREAL	0.02
OTHER/MIXED RACE, AGE 14-19	RICE KRISPIES TREATS CEREAL, KELLOGG'S	0.02
OTHER/MIXED RACE, AGE 14-19	SPECIAL K FRUIT & YOGURT	0.02
OTHER/MIXED RACE, AGE 14-19	CORN POPS CEREAL	0.02
OTHER/MIXED RACE, AGE 14-19	OTHER	0.08

POPULATION	CEREAL NAME	WEIGHT
NON-HISPANIC WHITE, AGE 20-30	HONEY NUT CHEERIOS	0.08
NON-HISPANIC WHITE, AGE 20-30	HONEY BUNCHES OF OATS W/ ALMONDS, POST	0.05
NON-HISPANIC WHITE, AGE 20-30	FRUITY PEBBLES CEREAL	0.05
NON-HISPANIC WHITE, AGE 20-30	RAISIN BRAN, KELLOGG'S	0.04
NON-HISPANIC WHITE, AGE 20-30	FROSTED MINI-WHEATS CEREAL (INCL ALL FLAVORS)	0.04
NON-HISPANIC WHITE, AGE 20-30	CHEERIOS	0.04
NON-HISPANIC WHITE, AGE 20-30	FROSTED FLAKES, KELLOGG'S	0.03
NON-HISPANIC WHITE, AGE 20-30	CINNAMON TOAST CRUNCH CEREAL	0.03
NON-HISPANIC WHITE, AGE 20-30	GOLDEN GRAHAMS CEREAL	0.03
NON-HISPANIC WHITE, AGE 20-30	COCOA PEBBLES CEREAL	0.03
NON-HISPANIC WHITE, AGE 20-30	RAISIN BRAN CEREAL, NFS	0.02
NON-HISPANIC WHITE, AGE 20-30	GRANOLA, LOWFAT, KELLOGG'S	0.02
NON-HISPANIC WHITE, AGE 20-30	SPECIAL K FRUIT & YOGURT	0.02
NON-HISPANIC WHITE, AGE 20-30	FROOT LOOPS CEREAL	0.02
NON-HISPANIC WHITE, AGE 20-30	CEREAL, READY-TO-EAT, NFS	0.02
NON-HISPANIC WHITE, AGE 20-30	GRANOLA, NFS	0.02
NON-HISPANIC WHITE, AGE 20-30	REESE'S PEANUT BUTTER PUFFS CEREAL	0.02
NON-HISPANIC WHITE, AGE 20-30	CAP'N CRUNCH CEREAL	0.02

NON-HISPANIC WHITE, AGE 20-30	RICE KRISPIES, KELLOGG'S	0.02
NON-HISPANIC WHITE, AGE 20-30	APPLE JACKS CEREAL	0.02
NON-HISPANIC WHITE, AGE 20-30	OTHER	0.39

POPULATION	CEREAL NAME	WEIGHT
NON-HISPANIC BLACK, AGE 20-30	FROSTED FLAKES, KELLOGG'S	0.09
NON-HISPANIC BLACK, AGE 20-30	CINNAMON TOAST CRUNCH CEREAL	0.08
NON-HISPANIC BLACK, AGE 20-30	FRUITY PEBBLES CEREAL	0.07
NON-HISPANIC BLACK, AGE 20-30	HONEY BUNCHES OF OATS CEREAL	0.07
NON-HISPANIC BLACK, AGE 20-30	HONEY NUT CHEERIOS	0.05
NON-HISPANIC BLACK, AGE 20-30	CORN FLAKES, KELLOGG'S	0.05
NON-HISPANIC BLACK, AGE 20-30	CORN POPS CEREAL	0.04
NON-HISPANIC BLACK, AGE 20-30	FROSTED MINI-WHEATS CEREAL (INCL ALL FLAVORS)	0.03
NON-HISPANIC BLACK, AGE 20-30	HONEYCOMB CEREAL, PLAIN	0.03
NON-HISPANIC BLACK, AGE 20-30	CHEERIOS	0.03
NON-HISPANIC BLACK, AGE 20-30	RAISIN BRAN, KELLOGG'S	0.03
NON-HISPANIC BLACK, AGE 20-30	CAP'N CRUNCH'S CRUNCH BERRIES CEREAL	0.03
NON-HISPANIC BLACK, AGE 20-30	GOLDEN GRAHAMS CEREAL	0.03
NON-HISPANIC BLACK, AGE 20-30	CAP'N CRUNCH CEREAL	0.03
NON-HISPANIC BLACK, AGE 20-30	FROOT LOOPS CEREAL	0.02
NON-HISPANIC BLACK, AGE 20-30	REESE'S PEANUT BUTTER PUFFS CEREAL	0.02
NON-HISPANIC BLACK, AGE 20-30	LUCKY CHARMS CEREAL	0.02
NON-HISPANIC BLACK, AGE 20-30	RICE KRISPIES, KELLOGG'S	0.02
NON-HISPANIC BLACK, AGE 20-30	HONEY BUNCHES OF OATS W/ ALMONDS, POST	0.02
NON-HISPANIC BLACK, AGE 20-30	RAISIN BRAN CRUNCH, KELLOGG'S	0.02
NON-HISPANIC BLACK, AGE 20-30	OTHER	0.23

POPULATION	CEREAL NAME	WEIGHT
MEXICAN-AMERICAN, AGE 20-30	CORN FLAKES, KELLOGG'S	0.09
MEXICAN-AMERICAN, AGE 20-30	HONEY NUT CHEERIOS	0.06
MEXICAN-AMERICAN, AGE 20-30	LUCKY CHARMS CEREAL	0.05
MEXICAN-AMERICAN, AGE 20-30	SPECIAL K RED BERRIES	0.05
MEXICAN-AMERICAN, AGE 20-30	FROSTED MINI-WHEATS CEREAL (INCL ALL FLAVORS)	0.05
MEXICAN-AMERICAN, AGE 20-30	CHEERIOS	0.05
MEXICAN-AMERICAN, AGE 20-30	COCOA PUFFS CEREAL	0.04
MEXICAN-AMERICAN, AGE 20-30	CINNAMON TOAST CRUNCH CEREAL	0.04
MEXICAN-AMERICAN, AGE 20-30	HONEY BUNCHES OF OATS W/ ALMONDS, POST	0.04
MEXICAN-AMERICAN, AGE 20-30	FROSTED FLAKES, KELLOGG'S	0.03
MEXICAN-AMERICAN, AGE 20-30	CEREAL, READY-TO-EAT, NFS	0.03
MEXICAN-AMERICAN, AGE 20-30	RAISIN BRAN CEREAL, NFS	0.03
MEXICAN-AMERICAN, AGE 20-30	HONEY BUNCHES OF OATS CEREAL	0.03
MEXICAN-AMERICAN, AGE 20-30	GRANOLA, NFS	0.02
MEXICAN-AMERICAN, AGE 20-30	FROOT LOOPS CEREAL	0.02
MEXICAN-AMERICAN, AGE 20-30	RAISIN BRAN, TOTAL	0.02

MEXICAN-AMERICAN, AGE 20-30	COCOA KRISPIES CEREAL	0.02
MEXICAN-AMERICAN, AGE 20-30	CORN FLAKES, NFS (INCLUDE STORE BRANDS)	0.02
MEXICAN-AMERICAN, AGE 20-30	FRUITY PEBBLES CEREAL	0.02
MEXICAN-AMERICAN, AGE 20-30	RAISIN BRAN, KELLOGG'S	0.02
MEXICAN-AMERICAN, AGE 20-30	OTHER	0.24

POPULATION	CEREAL NAME	WEIGHT
OTHER HISPANIC, AGE 20-30	FROSTED FLAKES, KELLOGG'S	0.13
OTHER HISPANIC, AGE 20-30	HONEY NUT CHEERIOS	0.09
OTHER HISPANIC, AGE 20-30	CORN FLAKES, KELLOGG'S	0.09
OTHER HISPANIC, AGE 20-30	FRUITY PEBBLES CEREAL	0.06
OTHER HISPANIC, AGE 20-30	FROSTED MINI-WHEATS CEREAL (INCL ALL FLAVORS)	0.06
OTHER HISPANIC, AGE 20-30	HONEY BUNCHES OF OATS CEREAL	0.05
OTHER HISPANIC, AGE 20-30	SPECIAL K CEREAL	0.04
OTHER HISPANIC, AGE 20-30	CORN POPS CEREAL	0.03
OTHER HISPANIC, AGE 20-30	SPECIAL K VANILLA ALMOND	0.03
OTHER HISPANIC, AGE 20-30	GRANOLA, NFS	0.02
OTHER HISPANIC, AGE 20-30	KASHI GOLEAN CRUNCH	0.02
OTHER HISPANIC, AGE 20-30	CAP'N CRUNCH'S PEANUT BUTTER CRUNCH CEREAL	0.02
OTHER HISPANIC, AGE 20-30	COCOA PEBBLES CEREAL	0.02
OTHER HISPANIC, AGE 20-30	APPLE JACKS CEREAL	0.02
OTHER HISPANIC, AGE 20-30	RAISIN BRAN CEREAL, NFS	0.02
OTHER HISPANIC, AGE 20-30	HONEY BUNCHES OF OATS W/ ALMONDS, POST	0.02
OTHER HISPANIC, AGE 20-30	SPECIAL K RED BERRIES	0.02
OTHER HISPANIC, AGE 20-30	NATURE VALLEY GRANOLA, W/ FRUIT & NUTS	0.02
OTHER HISPANIC, AGE 20-30	TRIX CEREAL	0.01
OTHER HISPANIC, AGE 20-30	LUCKY CHARMS CEREAL	0.01
OTHER HISPANIC, AGE 20-30	OTHER	0.20

POPULATION	CEREAL NAME	WEIGHT
OTHER/MIXED RACE, AGE 20-30	HONEY BUNCHES OF OATS W/ ALMONDS, POST	0.16
OTHER/MIXED RACE, AGE 20-30	CHEERIOS	0.11
OTHER/MIXED RACE, AGE 20-30	REESE'S PEANUT BUTTER PUFFS CEREAL	0.08
OTHER/MIXED RACE, AGE 20-30	HONEY BUNCHES OF OATS CEREAL	0.07
OTHER/MIXED RACE, AGE 20-30	HONEY BUNCHES OF OATS WITH VANILLA CLUSTERS, POST	0.07
OTHER/MIXED RACE, AGE 20-30	KASHI GOLEAN CRUNCH HONEY ALMOND FLAX	0.07
OTHER/MIXED RACE, AGE 20-30	RICE CHEX CEREAL	0.07
OTHER/MIXED RACE, AGE 20-30	FROSTED FLAKES, KELLOGG'S	0.06
OTHER/MIXED RACE, AGE 20-30	SPECIAL K RED BERRIES	0.05
OTHER/MIXED RACE, AGE 20-30	HONEY SMACKS, KELLOGG'S	0.04
OTHER/MIXED RACE, AGE 20-30	FROOT LOOPS CEREAL	0.04
OTHER/MIXED RACE, AGE 20-30	SPECIAL K FRUIT & YOGURT	0.03
OTHER/MIXED RACE, AGE 20-30	CAP'N CRUNCH'S PEANUT BUTTER CRUNCH CEREAL	0.02
OTHER/MIXED RACE, AGE 20-30	GRANOLA, NFS	0.02
OTHER/MIXED RACE, AGE 20-30	LUCKY CHARMS CEREAL	0.02
OTHER/MIXED RACE, AGE 20-30	CINNAMON TOAST CRUNCH CEREAL	0.02
OTHER/MIXED RACE, AGE 20-30	SPECIAL K VANILLA ALMOND	0.02
OTHER/MIXED RACE, AGE 20-30	CORN FLAKES, NFS (INCLUDE STORE BRANDS)	0.01
OTHER/MIXED RACE, AGE 20-30	GRANOLA, LOWFAT, KELLOGG'S	0.01
OTHER/MIXED RACE, AGE 20-30	MULTIGRAIN CHEERIOS	0.01
OTHER/MIXED RACE, AGE 20-30	OTHER	0.00

POPULATION	CEREAL NAME	WEIGHT
NON-HISPANIC WHITE, AGE 31-50	CHEERIOS	0.07
NON-HISPANIC WHITE, AGE 31-50	FROSTED MINI-WHEATS CEREAL (INCL ALL FLAVORS)	0.06
NON-HISPANIC WHITE, AGE 31-50	GRANOLA, NFS	0.06
NON-HISPANIC WHITE, AGE 31-50	HONEY NUT CHEERIOS	0.06
NON-HISPANIC WHITE, AGE 31-50	RAISIN BRAN, KELLOGG'S	0.04
NON-HISPANIC WHITE, AGE 31-50	RAISIN BRAN CEREAL, NFS	0.03
NON-HISPANIC WHITE, AGE 31-50	FROSTED FLAKES, KELLOGG'S	0.03
NON-HISPANIC WHITE, AGE 31-50	LIFE CEREAL (PLAIN & CINNAMON)	0.02
NON-HISPANIC WHITE, AGE 31-50	CAP'N CRUNCH CEREAL	0.02
NON-HISPANIC WHITE, AGE 31-50	GRANOLA, HOMEMADE	0.02
NON-HISPANIC WHITE, AGE 31-50	HONEY BUNCHES OF OATS CEREAL	0.02
NON-HISPANIC WHITE, AGE 31-50	RAISIN BRAN CEREAL, POST	0.02
NON-HISPANIC WHITE, AGE 31-50	RAISIN BRAN CRUNCH, KELLOGG'S	0.02
NON-HISPANIC WHITE, AGE 31-50	COCOA PEBBLES CEREAL	0.02
NON-HISPANIC WHITE, AGE 31-50	CORN FLAKES, KELLOGG'S	0.02
NON-HISPANIC WHITE, AGE 31-50	HONEY BUNCHES OF OATS W/ ALMONDS, POST	0.02
NON-HISPANIC WHITE, AGE 31-50	SPECIAL K RED BERRIES	0.02
NON-HISPANIC WHITE, AGE 31-50	QUAKER OATMEAL SQUARES CEREAL (FORMERLY QUAKER OAT SQUARES)	0.02
NON-HISPANIC WHITE, AGE 31-50	CEREAL, READY-TO-EAT, NFS	0.01

NON-HISPANIC WHITE, AGE 31-50	FROOT LOOPS CEREAL	0.01
NON-HISPANIC WHITE, AGE 31-50	OTHER	0.40

POPULATION	CEREAL NAME	WEIGHT
NON-HISPANIC BLACK, AGE 31-50	FROSTED FLAKES, KELLOGG'S	0.15
NON-HISPANIC BLACK, AGE 31-50	RAISIN BRAN, KELLOGG'S	0.14
NON-HISPANIC BLACK, AGE 31-50	HONEY NUT CHEERIOS	0.07
NON-HISPANIC BLACK, AGE 31-50	CORN FLAKES, KELLOGG'S	0.06
NON-HISPANIC BLACK, AGE 31-50	CHEERIOS	0.05
NON-HISPANIC BLACK, AGE 31-50	CORN POPS CEREAL	0.03
NON-HISPANIC BLACK, AGE 31-50	COCOA PUFFS CEREAL	0.03
NON-HISPANIC BLACK, AGE 31-50	CAP'N CRUNCH'S CRUNCH BERRIES CEREAL	0.03
NON-HISPANIC BLACK, AGE 31-50	FRUITY PEBBLES CEREAL	0.02
NON-HISPANIC BLACK, AGE 31-50	HONEY BUNCHES OF OATS CEREAL	0.02
NON-HISPANIC BLACK, AGE 31-50	GREAT GRAINS, RAISIN, DATE, & PECAN,WHOLE GRAIN CEREAL, POST	0.02
NON-HISPANIC BLACK, AGE 31-50	RAISIN BRAN CRUNCH, KELLOGG'S	0.02
NON-HISPANIC BLACK, AGE 31-50	APPLE JACKS CEREAL	0.02
NON-HISPANIC BLACK, AGE 31-50	HONEY SMACKS, KELLOGG'S	0.02
NON-HISPANIC BLACK, AGE 31-50	GRANOLA, NFS	0.02
NON-HISPANIC BLACK, AGE 31-50	FROSTED MINI-WHEATS CEREAL (INCL ALL FLAVORS)	0.02
NON-HISPANIC BLACK, AGE 31-50	TOASTED OAT CEREAL	0.02
NON-HISPANIC BLACK, AGE 31-50	SPECIAL K RED BERRIES	0.01
NON-HISPANIC BLACK, AGE 31-50	FROOT LOOPS CEREAL	0.01
NON-HISPANIC BLACK, AGE 31-50	RICE KRISPIES, KELLOGG'S	0.01
NON-HISPANIC BLACK, AGE 31-50	OTHER	0.22

POPULATION	CEREAL NAME	WEIGHT
MEXICAN-AMERICAN, AGE 31-50	CORN FLAKES, KELLOGG'S	0.10
MEXICAN-AMERICAN, AGE 31-50	HONEY BUNCHES OF OATS CEREAL	0.10
MEXICAN-AMERICAN, AGE 31-50	HONEY BUNCHES OF OATS W/ ALMONDS, POST	0.09
MEXICAN-AMERICAN, AGE 31-50	RAISIN BRAN, KELLOGG'S	0.08
MEXICAN-AMERICAN, AGE 31-50	FROSTED FLAKES, KELLOGG'S	0.05
MEXICAN-AMERICAN, AGE 31-50	RAISIN BRAN CEREAL, NFS	0.05
MEXICAN-AMERICAN, AGE 31-50	FROSTED MINI-WHEATS CEREAL (INCL ALL FLAVORS)	0.05
MEXICAN-AMERICAN, AGE 31-50	CHEERIOS	0.04
MEXICAN-AMERICAN, AGE 31-50	GRANOLA, NFS	0.04
MEXICAN-AMERICAN, AGE 31-50	CEREAL, READY-TO-EAT, NFS	0.04
MEXICAN-AMERICAN, AGE 31-50	LIFE CEREAL (PLAIN & CINNAMON)	0.03
MEXICAN-AMERICAN, AGE 31-50	HONEY NUT CHEERIOS	0.03
MEXICAN-AMERICAN, AGE 31-50	FROOT LOOPS CEREAL	0.03
MEXICAN-AMERICAN, AGE 31-50	HONEY SMACKS, KELLOGG'S	0.02
MEXICAN-AMERICAN, AGE 31-50	SPECIAL K CEREAL	0.02
MEXICAN-AMERICAN, AGE 31-50	RAISIN NUT BRAN CEREAL	0.02
MEXICAN-AMERICAN, AGE 31-50	FRUITY PEBBLES CEREAL	0.01

MEXICAN-AMERICAN, AGE 31-50	CORN POPS CEREAL	0.01
MEXICAN-AMERICAN, AGE 31-50	CORN FLAKES, NFS (INCLUDE STORE BRANDS)	0.01
MEXICAN-AMERICAN, AGE 31-50	FROSTED CORN FLAKES, NFS	0.01
MEXICAN-AMERICAN, AGE 31-50	OTHER	0.17

POPULATION	CEREAL NAME	WEIGHT
OTHER HISPANIC, AGE 31-50	FROSTED FLAKES, KELLOGG'S	0.09
OTHER HISPANIC, AGE 31-50	HONEY NUT CHEERIOS	0.09
OTHER HISPANIC, AGE 31-50	CORN FLAKES, KELLOGG'S	0.07
OTHER HISPANIC, AGE 31-50	RAISIN BRAN CEREAL, NFS	0.06
OTHER HISPANIC, AGE 31-50	GRAPE-NUTS CEREAL	0.06
OTHER HISPANIC, AGE 31-50	RAISIN BRAN CEREAL, POST	0.05
OTHER HISPANIC, AGE 31-50	CEREAL, READY-TO-EAT, NFS	0.04
OTHER HISPANIC, AGE 31-50	FROSTED MINI-WHEATS CEREAL (INCL ALL FLAVORS)	0.04
OTHER HISPANIC, AGE 31-50	RAISIN BRAN, KELLOGG'S	0.04
OTHER HISPANIC, AGE 31-50	HONEY BUNCHES OF OATS CEREAL	0.04
OTHER HISPANIC, AGE 31-50	APPLE JACKS CEREAL	0.03
OTHER HISPANIC, AGE 31-50	OATMEAL CRISP, RAISIN	0.03
OTHER HISPANIC, AGE 31-50	CINNAMON TOAST CRUNCH CEREAL	0.03
OTHER HISPANIC, AGE 31-50	CHEERIOS	0.03
OTHER HISPANIC, AGE 31-50	SPECIAL K RED BERRIES	0.03
OTHER HISPANIC, AGE 31-50	GRANOLA, NFS	0.02
OTHER HISPANIC, AGE 31-50	SPECIAL K CEREAL	0.02
OTHER HISPANIC, AGE 31-50	HONEY NUT CHEX CEREAL	0.02
OTHER HISPANIC, AGE 31-50	HONEY BUNCHES OF OATS W/ ALMONDS, POST	0.02
OTHER HISPANIC, AGE 31-50	FROSTED CORN FLAKES, NFS	0.02
OTHER HISPANIC, AGE 31-50	OTHER	0.18

POPULATION	CEREAL NAME	WEIGHT
OTHER/MIXED RACE, AGE 31-50	HONEY NUT CHEERIOS	0.12
OTHER/MIXED RACE, AGE 31-50	CEREAL, READY-TO-EAT, NFS	0.10
OTHER/MIXED RACE, AGE 31-50	FROSTED MINI-WHEATS CEREAL (INCL ALL FLAVORS)	0.07
OTHER/MIXED RACE, AGE 31-50	HONEYCOMB CEREAL, PLAIN	0.06
OTHER/MIXED RACE, AGE 31-50	HONEY BUNCHES OF OATS CEREAL	0.06
OTHER/MIXED RACE, AGE 31-50	MULTIGRAIN CHEERIOS	0.04
OTHER/MIXED RACE, AGE 31-50	CAP'N CRUNCH'S CRUNCH BERRIES CEREAL	0.04
OTHER/MIXED RACE, AGE 31-50	YOGURT BURST CHEERIOS	0.04
OTHER/MIXED RACE, AGE 31-50	HONEY BUNCHES OF OATS W/ ALMONDS, POST	0.04
OTHER/MIXED RACE, AGE 31-50	TOTAL CEREAL	0.03
OTHER/MIXED RACE, AGE 31-50	RAISIN BRAN, KELLOGG'S	0.03
OTHER/MIXED RACE, AGE 31-50	CORN POPS CEREAL	0.03
OTHER/MIXED RACE, AGE 31-50	MUESLIX CEREAL, NFS	0.03
OTHER/MIXED RACE, AGE 31-50	SPECIAL K CINNAMON PECAN, KELLOGG'S	0.03

OTHER/MIXED RACE, AGE 31-50	SPECIAL K RED BERRIES	0.03
OTHER/MIXED RACE, AGE 31-50	CAP'N CRUNCH'S PEANUT BUTTER CRUNCH CEREAL	0.02
OTHER/MIXED RACE, AGE 31-50	OPTIMUM SLIM, NATURE'S PATH	0.02
OTHER/MIXED RACE, AGE 31-50	COOKIE-CRISP CEREAL (INCLUDE ALL FLAVORS)	0.02
OTHER/MIXED RACE, AGE 31-50	PUFFED RICE CEREAL	0.02
OTHER/MIXED RACE, AGE 31-50	GRANOLA, NFS	0.02
OTHER/MIXED RACE, AGE 31-50	OTHER	0.14

POPULATION	CEREAL NAME	WEIGHT
NON-HISPANIC WHITE, AGE 51-70	CHEERIOS	0.09
NON-HISPANIC WHITE, AGE 51-70	HONEY NUT CHEERIOS	0.07
NON-HISPANIC WHITE, AGE 51-70	FROSTED MINI-WHEATS CEREAL (INCL ALL FLAVORS)	0.06
NON-HISPANIC WHITE, AGE 51-70	RAISIN BRAN, KELLOGG'S	0.05
NON-HISPANIC WHITE, AGE 51-70	GRANOLA, NFS	0.04
NON-HISPANIC WHITE, AGE 51-70	HONEY BUNCHES OF OATS W/ ALMONDS, POST	0.04
NON-HISPANIC WHITE, AGE 51-70	SHREDDED WHEAT, 100%	0.03
NON-HISPANIC WHITE, AGE 51-70	BRAN FLAKES CEREAL, NFS (FORMERLY 40% BRAN FLAKES, NFS)	0.03
NON-HISPANIC WHITE, AGE 51-70	GRAPE-NUTS CEREAL	0.02
NON-HISPANIC WHITE, AGE 51-70	FROSTED FLAKES, KELLOGG'S	0.02
NON-HISPANIC WHITE, AGE 51-70	HONEY BUNCHES OF OATS CEREAL	0.02
NON-HISPANIC WHITE, AGE 51-70	RICE KRISPIES, KELLOGG'S	0.02
NON-HISPANIC WHITE, AGE 51-70	SPECIAL K CEREAL	0.02
NON-HISPANIC WHITE, AGE 51-70	RAISIN BRAN CEREAL, NFS	0.02
NON-HISPANIC WHITE, AGE 51-70	CORN FLAKES, KELLOGG'S	0.02
NON-HISPANIC WHITE, AGE 51-70	RAISIN BRAN CEREAL, POST	0.02
NON-HISPANIC WHITE, AGE 51-70	QUAKER OATMEAL SQUARES CEREAL (FORMERLY QUAKER OAT SQUARES)	0.01
NON-HISPANIC WHITE, AGE 51-70	SPECIAL K RED BERRIES	0.01
NON-HISPANIC WHITE, AGE 51-70	MUESLIX CEREAL, NFS	0.01
NON-HISPANIC WHITE, AGE 51-70	KASHI GOLEAN	0.01
NON-HISPANIC WHITE, AGE 51-70	OTHER	0.38

POPULATION	CEREAL NAME	WEIGHT
NON-HISPANIC BLACK, AGE 51-70	HONEY NUT CHEERIOS	0.11
NON-HISPANIC BLACK, AGE 51-70	RAISIN BRAN, KELLOGG'S	0.11
NON-HISPANIC BLACK, AGE 51-70	CHEERIOS	0.08
NON-HISPANIC BLACK, AGE 51-70	CORN FLAKES, KELLOGG'S	0.08
NON-HISPANIC BLACK, AGE 51-70	FROSTED FLAKES, KELLOGG'S	0.07
NON-HISPANIC BLACK, AGE 51-70	RAISIN BRAN CEREAL, POST	0.06
NON-HISPANIC BLACK, AGE 51-70	GRANOLA, NFS	0.04
NON-HISPANIC BLACK, AGE 51-70	RICE KRISPIES, KELLOGG'S	0.04
NON-HISPANIC BLACK, AGE 51-70	RAISIN BRAN CEREAL, NFS	0.03
NON-HISPANIC BLACK, AGE 51-70	SPECIAL K CEREAL	0.02
NON-HISPANIC BLACK, AGE 51-70	CORN POPS CEREAL	0.02

NON-HISPANIC BLACK, AGE 51-70	HONEY BUNCHES OF OATS W/ ALMONDS, POST	0.02
NON-HISPANIC BLACK, AGE 51-70	FROSTED MINI-WHEATS CEREAL (INCL ALL FLAVORS)	0.02
NON-HISPANIC BLACK, AGE 51-70	LIFE CEREAL (PLAIN & CINNAMON)	0.02
NON-HISPANIC BLACK, AGE 51-70	BANANA NUT CRUNCH CEREAL (POST)	0.01
NON-HISPANIC BLACK, AGE 51-70	HONEY BUNCHES OF OATS CEREAL	0.01
NON-HISPANIC BLACK, AGE 51-70	FIBER ONE CEREAL	0.01
NON-HISPANIC BLACK, AGE 51-70	CORN CHEX CEREAL	0.01
NON-HISPANIC BLACK, AGE 51-70	HONEY SMACKS, KELLOGG'S	0.01
NON-HISPANIC BLACK, AGE 51-70	100 % NATURAL CEREAL, W/ OATS,HONEY & RAISINS,QUAKER	0.01
NON-HISPANIC BLACK, AGE 51-70	OTHER	0.20

POPULATION	CEREAL NAME	WEIGHT
MEXICAN-AMERICAN, AGE 51-70	CORN FLAKES, KELLOGG'S	0.18
MEXICAN-AMERICAN, AGE 51-70	CHEERIOS	0.10
MEXICAN-AMERICAN, AGE 51-70	HONEY NUT CHEERIOS	0.08
MEXICAN-AMERICAN, AGE 51-70	GRANOLA, NFS	0.07
MEXICAN-AMERICAN, AGE 51-70	HONEY BUNCHES OF OATS CEREAL	0.06
MEXICAN-AMERICAN, AGE 51-70	FROSTED FLAKES, KELLOGG'S	0.04
MEXICAN-AMERICAN, AGE 51-70	RAISIN BRAN, KELLOGG'S	0.04
MEXICAN-AMERICAN, AGE 51-70	SPECIAL K RED BERRIES	0.04
MEXICAN-AMERICAN, AGE 51-70	CORN FLAKES, NFS (INCLUDE STORE BRANDS)	0.04
MEXICAN-AMERICAN, AGE 51-70	HONEY BUNCHES OF OATS W/ ALMONDS, POST	0.03
MEXICAN-AMERICAN, AGE 51-70	FROSTED MINI-WHEATS CEREAL (INCL ALL FLAVORS)	0.03
MEXICAN-AMERICAN, AGE 51-70	RICE KRISPIES, KELLOGG'S	0.03
MEXICAN-AMERICAN, AGE 51-70	RAISIN BRAN CEREAL, NFS	0.03
MEXICAN-AMERICAN, AGE 51-70	CEREAL, READY-TO-EAT, NFS	0.03
MEXICAN-AMERICAN, AGE 51-70	SPECIAL K CEREAL	0.02
MEXICAN-AMERICAN, AGE 51-70	SPECIAL K VANILLA ALMOND	0.02
MEXICAN-AMERICAN, AGE 51-70	LIFE CEREAL (PLAIN & CINNAMON)	0.02
MEXICAN-AMERICAN, AGE 51-70	WHEAT CHEX CEREAL	0.01
MEXICAN-AMERICAN, AGE 51-70	TOTAL CEREAL	0.01
MEXICAN-AMERICAN, AGE 51-70	BRAN FLAKES CEREAL, NFS (FORMERLY 40% BRAN FLAKES, NFS)	0.01
MEXICAN-AMERICAN, AGE 51-70	OTHER	0.12

POPULATION	CEREAL NAME	WEIGHT
OTHER HISPANIC, AGE 51-70	CORN FLAKES, KELLOGG'S	0.17
OTHER HISPANIC, AGE 51-70	CHEERIOS	0.12
OTHER HISPANIC, AGE 51-70	HONEY NUT CHEERIOS	0.10
OTHER HISPANIC, AGE 51-70	FROSTED FLAKES, KELLOGG'S	0.07
OTHER HISPANIC, AGE 51-70	SPECIAL K CEREAL	0.05
OTHER HISPANIC, AGE 51-70	CEREAL, READY-TO-EAT, NFS	0.05
OTHER HISPANIC, AGE 51-70	FROSTED MINI-WHEATS CEREAL (INCL ALL FLAVORS)	0.04
OTHER HISPANIC, AGE 51-70	RAISIN BRAN, KELLOGG'S	0.03

OTHER HISPANIC, AGE 51-70	HONEY BUNCHES OF OATS W/ ALMONDS, POST	0.03
OTHER HISPANIC, AGE 51-70	GRANOLA, NFS	0.03
OTHER HISPANIC, AGE 51-70	ALL-BRAN CEREAL	0.02
OTHER HISPANIC, AGE 51-70	HONEY NUT CLUSTERS CEREAL	0.02
OTHER HISPANIC, AGE 51-70	KASHI GOLEAN	0.02
OTHER HISPANIC, AGE 51-70	CORN FLAKES, NFS (INCLUDE STORE BRANDS)	0.02
OTHER HISPANIC, AGE 51-70	FROSTED CORN FLAKES, NFS	0.02
OTHER HISPANIC, AGE 51-70	FRUITY CHEERIOS	0.02
OTHER HISPANIC, AGE 51-70	SPECIAL K RED BERRIES	0.02
OTHER HISPANIC, AGE 51-70	HONEY BUNCHES OF OATS CEREAL	0.02
OTHER HISPANIC, AGE 51-70	RICE KRISPIES, KELLOGG'S	0.02
OTHER HISPANIC, AGE 51-70	SHREDDED WHEAT, 100%	0.01
OTHER HISPANIC, AGE 51-70	OTHER	0.12

POPULATION	CEREAL NAME	WEIGHT
OTHER/MIXED RACE, AGE 51-70	HONEY NUT CHEERIOS	0.15
OTHER/MIXED RACE, AGE 51-70	FROSTED MINI-WHEATS CEREAL (INCL ALL FLAVORS)	0.08
OTHER/MIXED RACE, AGE 51-70	HONEY BUNCHES OF OATS W/ ALMONDS, POST	0.07
OTHER/MIXED RACE, AGE 51-70	FROSTED FLAKES, KELLOGG'S	0.06
OTHER/MIXED RACE, AGE 51-70	CHEERIOS	0.06
OTHER/MIXED RACE, AGE 51-70	ALPEN CEREAL	0.06
OTHER/MIXED RACE, AGE 51-70	GRANOLA, NFS	0.05
OTHER/MIXED RACE, AGE 51-70	WHEATIES CEREAL	0.04
OTHER/MIXED RACE, AGE 51-70	CAP'N CRUNCH CEREAL	0.04
OTHER/MIXED RACE, AGE 51-70	FIBER ONE CEREAL	0.04
OTHER/MIXED RACE, AGE 51-70	KASHI HEART TO HEART HONEY TOASTED OAT	0.04
OTHER/MIXED RACE, AGE 51-70	LIFE CEREAL (PLAIN & CINNAMON)	0.03
OTHER/MIXED RACE, AGE 51-70	OATMEAL CRISP W/ ALMONDS CEREAL	0.03
OTHER/MIXED RACE, AGE 51-70	SPECIAL K RED BERRIES	0.03
OTHER/MIXED RACE, AGE 51-70	OAT CEREAL, NFS	0.03
OTHER/MIXED RACE, AGE 51-70	RAISIN BRAN, KELLOGG'S	0.03
OTHER/MIXED RACE, AGE 51-70	RAISIN BRAN CEREAL, NFS	0.02
OTHER/MIXED RACE, AGE 51-70	BRAN FLAKES CEREAL, NFS (FORMERLY 40% BRAN FLAKES, NFS)	0.02
OTHER/MIXED RACE, AGE 51-70	GOLDEN CRISP CEREAL	0.02
OTHER/MIXED RACE, AGE 51-70	CEREAL, READY-TO-EAT, NFS	0.02
OTHER/MIXED RACE, AGE 51-70	OTHER	0.08

POPULATION	CEREAL NAME	WEIGHT
NON-HISPANIC WHITE, AGE 71+	CHEERIOS	0.10
NON-HISPANIC WHITE, AGE 71+	HONEY NUT CHEERIOS	0.08
NON-HISPANIC WHITE, AGE 71+	RAISIN BRAN, KELLOGG'S	0.05
NON-HISPANIC WHITE, AGE 71+	SHREDDED WHEAT, 100%	0.05
NON-HISPANIC WHITE, AGE 71+	FROSTED MINI-WHEATS CEREAL (INCL ALL FLAVORS)	0.04

NON-HISPANIC WHITE, AGE 71+	RAISIN BRAN CEREAL, POST	0.04
NON-HISPANIC WHITE, AGE 71+	RAISIN BRAN CEREAL, NFS	0.03
NON-HISPANIC WHITE, AGE 71+	BRAN FLAKES CEREAL, NFS (FORMERLY 40% BRAN FLAKES, NFS)	0.03
NON-HISPANIC WHITE, AGE 71+	CORN FLAKES, KELLOGG'S	0.03
NON-HISPANIC WHITE, AGE 71+	CEREAL, READY-TO-EAT, NFS	0.03
NON-HISPANIC WHITE, AGE 71+	HONEY BUNCHES OF OATS CEREAL	0.03
NON-HISPANIC WHITE, AGE 71+	GRANOLA, NFS	0.03
NON-HISPANIC WHITE, AGE 71+	RICE KRISPIES, KELLOGG'S	0.02
NON-HISPANIC WHITE, AGE 71+	SPECIAL K CEREAL	0.02
NON-HISPANIC WHITE, AGE 71+	FROSTED FLAKES, KELLOGG'S	0.02
NON-HISPANIC WHITE, AGE 71+	FIBER ONE CEREAL	0.02
NON-HISPANIC WHITE, AGE 71+	TOTAL CEREAL	0.02
NON-HISPANIC WHITE, AGE 71+	MULTIGRAIN CHEERIOS	0.02
NON-HISPANIC WHITE, AGE 71+	WHEATIES CEREAL	0.02
NON-HISPANIC WHITE, AGE 71+	GRAPE-NUTS CEREAL	0.02
NON-HISPANIC WHITE, AGE 71+	OTHER	0.34

POPULATION	CEREAL NAME	WEIGHT
NON-HISPANIC BLACK, AGE 71+	CORN FLAKES, KELLOGG'S	0.20
NON-HISPANIC BLACK, AGE 71+	HONEY NUT CHEERIOS	0.10
NON-HISPANIC BLACK, AGE 71+	RAISIN BRAN, KELLOGG'S	0.08
NON-HISPANIC BLACK, AGE 71+	FROSTED FLAKES, KELLOGG'S	0.08
NON-HISPANIC BLACK, AGE 71+	TOTAL CEREAL	0.06
NON-HISPANIC BLACK, AGE 71+	CHEERIOS	0.06
NON-HISPANIC BLACK, AGE 71+	RAISIN BRAN, TOTAL	0.05
NON-HISPANIC BLACK, AGE 71+	CEREAL, READY-TO-EAT, NFS	0.05
NON-HISPANIC BLACK, AGE 71+	OATMEAL CRISP W/ ALMONDS CEREAL	0.04
NON-HISPANIC BLACK, AGE 71+	SHREDDED WHEAT, 100%	0.04
NON-HISPANIC BLACK, AGE 71+	RICE KRISPIES, KELLOGG'S	0.03
NON-HISPANIC BLACK, AGE 71+	SPECIAL K CEREAL	0.03
NON-HISPANIC BLACK, AGE 71+	RAISIN BRAN CEREAL, NFS	0.02
NON-HISPANIC BLACK, AGE 71+	WEETABIX WHOLE WHEAT CEREAL	0.02
NON-HISPANIC BLACK, AGE 71+	CORN FLAKES, NFS (INCLUDE STORE BRANDS)	0.02
NON-HISPANIC BLACK, AGE 71+	SPECIAL K CINNAMON PECAN, KELLOGG'S	0.02
NON-HISPANIC BLACK, AGE 71+	RAISIN BRAN CEREAL, POST	0.01
NON-HISPANIC BLACK, AGE 71+	PUFFED RICE CEREAL	0.01
NON-HISPANIC BLACK, AGE 71+	CINNAMON TOAST CRUNCH CEREAL	0.01
NON-HISPANIC BLACK, AGE 71+	SPECIAL K FRUIT & YOGURT	0.01
NON-HISPANIC BLACK, AGE 71+	OTHER	0.05

POPULATION	CEREAL NAME	WEIGHT
MEXICAN-AMERICAN, AGE 71+	CEREAL, READY-TO-EAT, NFS	0.15
MEXICAN-AMERICAN, AGE 71+	HONEY BUNCHES OF OATS W/ ALMONDS, POST	0.12

MEXICAN-AMERICAN, AGE 71+	CORN FLAKES, KELLOGG'S	0.12
MEXICAN-AMERICAN, AGE 71+	CHEERIOS	0.08
MEXICAN-AMERICAN, AGE 71+	FROSTED CORN FLAKES, NFS	0.08
MEXICAN-AMERICAN, AGE 71+	GREAT GRAINS, RAISIN, DATE, & PECAN,WHOLE GRAIN CEREAL, POST	0.06
MEXICAN-AMERICAN, AGE 71+	HONEY BUNCHES OF OATS CEREAL	0.06
MEXICAN-AMERICAN, AGE 71+	HONEY NUT CHEERIOS	0.05
MEXICAN-AMERICAN, AGE 71+	CORN FLAKES, NFS (INCLUDE STORE BRANDS)	0.04
MEXICAN-AMERICAN, AGE 71+	RAISIN BRAN, KELLOGG'S	0.04
MEXICAN-AMERICAN, AGE 71+	FROSTED MINI-WHEATS CEREAL (INCL ALL FLAVORS)	0.03
MEXICAN-AMERICAN, AGE 71+	FROOT LOOPS CEREAL	0.03
MEXICAN-AMERICAN, AGE 71+	RAISIN BRAN CEREAL, NFS	0.03
MEXICAN-AMERICAN, AGE 71+	SPECIAL K CEREAL	0.02
MEXICAN-AMERICAN, AGE 71+	FROSTED WHEAT BITES	0.02
MEXICAN-AMERICAN, AGE 71+	LUCKY CHARMS CEREAL	0.02
MEXICAN-AMERICAN, AGE 71+	HONEY CRUNCH CORN FLAKES CEREAL, KELLOGG'S	0.01
MEXICAN-AMERICAN, AGE 71+	RICE KRISPIES, KELLOGG'S	0.01
MEXICAN-AMERICAN, AGE 71+	SHREDDED WHEAT, 100%	0.01
MEXICAN-AMERICAN, AGE 71+	FIBER ONE CEREAL	0.01
MEXICAN-AMERICAN, AGE 71+	OTHER	0.01

POPULATION	CEREAL NAME	WEIGHT
OTHER HISPANIC, AGE 71+	CORN FLAKES, KELLOGG'S	0.14
OTHER HISPANIC, AGE 71+	CINNAMON TOAST CRUNCH CEREAL	0.14
OTHER HISPANIC, AGE 71+	HONEY NUT CHEERIOS	0.10
OTHER HISPANIC, AGE 71+	RAISIN BRAN, KELLOGG'S	0.09
OTHER HISPANIC, AGE 71+	RAISIN BRAN CEREAL, POST	0.08
OTHER HISPANIC, AGE 71+	CHEERIOS	0.06
OTHER HISPANIC, AGE 71+	CORN FLAKES, NFS (INCLUDE STORE BRANDS)	0.06
OTHER HISPANIC, AGE 71+	SPECIAL K CEREAL	0.05
OTHER HISPANIC, AGE 71+	LIFE CEREAL (PLAIN & CINNAMON)	0.04
OTHER HISPANIC, AGE 71+	COCOA KRISPIES CEREAL	0.04
OTHER HISPANIC, AGE 71+	OATMEAL HONEY NUT HEAVEN, QUAKER	0.03
OTHER HISPANIC, AGE 71+	TOTAL CEREAL	0.03
OTHER HISPANIC, AGE 71+	FROSTED FLAKES, KELLOGG'S	0.03
OTHER HISPANIC, AGE 71+	KASHI, PUFFED	0.03
OTHER HISPANIC, AGE 71+	BLUEBERRY MORNING, POST	0.03
OTHER HISPANIC, AGE 71+	FROSTED MINI-WHEATS CEREAL (INCL ALL FLAVORS)	0.02
OTHER HISPANIC, AGE 71+	CEREAL, READY-TO-EAT, NFS	0.02
OTHER HISPANIC, AGE 71+	SHREDDED WHEAT, 100%	0.02
OTHER HISPANIC, AGE 71+	OTHER	0.00

POPULATION	CEREAL NAME	WEIGHT
OTHER/MIXED RACE, AGE 71+	CEREAL, READY-TO-EAT, NFS	0.28

OTHER/MIXED RACE, AGE 71+	RICE KRISPIES, KELLOGG'S	0.15
OTHER/MIXED RACE, AGE 71+	CHEERIOS	0.12
OTHER/MIXED RACE, AGE 71+	HONEY BUNCHES OF OATS W/ ALMONDS, POST	0.10
OTHER/MIXED RACE, AGE 71+	HONEY NUT CHEERIOS	0.09
OTHER/MIXED RACE, AGE 71+	BRAN FLAKES CEREAL, NFS (FORMERLY 40% BRAN FLAKES, NFS)	0.08
OTHER/MIXED RACE, AGE 71+	CORN FLAKES, KELLOGG'S	0.06
OTHER/MIXED RACE, AGE 71+	CORN FLAKES, NFS (INCLUDE STORE BRANDS)	0.04
OTHER/MIXED RACE, AGE 71+	RAISIN BRAN CEREAL, NFS	0.03
OTHER/MIXED RACE, AGE 71+	HONEY BUNCHES OF OATS CEREAL	0.02
OTHER/MIXED RACE, AGE 71+	OTHER	0.02

Supplemental Table S42. Sources of breakfast energy in the United States, 2007-2010

Food category	% of breakfast calories
Ready-to-eat cereals[a]	11.4
Milk, whole and reduced fat[a]	8.6
Breads and rolls	8.0
Eggs and egg mixed dishes	7.7
Doughnuts, sweet rolls, pastries	4.7
Cooked cereals	4.5
Pancakes, waffles, French toast	3.8
Citrus juices[a]	3.3
Sandwiches (single code)	2.6
Biscuits, muffins, quick breads	2.6
Frankfurters and sausages	2.2
Milk, lowfat and nonfat[a]	2.2
Jams, syrups, toppings	1.9
White potatoes, fried	1.8
Bananas	1.8
Sugars and honey[b]	1.7
Butter, margarine, other fats	1.6
Fruit juices, other than citrus[a]	1.5
Tortillas	1.5
Flavored milk[a]	1.5
Fruit drinks	1.4
Cheese	1.3
Bacon	1.3
Cream and cream substitutes[a]	1.3
Burritos, tacos, tamales	1.3
Soft drinks[a]	1.1
Cookies, brownies, sweet crackers	1.1
Nuts and seeds	1.0
Yogurt	1.0
Other[b,c]	14.2

[a] Foods and beverages not included in the replacement model.
[b] Some included in replacement model depending on whether it was consumed with food (included in replacement model) or with beverage (excluded from replacement model).
[c] Includes all foods/beverages contributing less than 1% of total breakfast calories.

Food categories were based on previous USDA analysis of sources of nutrients (http://www.cdc.gov/mmwr/preview/mmwrhtml/mm6105-table.htm).
